# Supplementary material for: Aromatic Diboronic Acids as Effective KPC/AmpC Inhibitors
Source: Molecules. 2023 Oct 31;28(21):7362. doi: 10.3390/molecules28217362 (PMC10648349; doi:10.3390/molecules28217362)
Supplement: Supplementary file 1 [file molecules-28-07362-s001.zip › molecules-2675999-supplementary.pdf]

Supplementary Materials

Aromatic Diboronic Acids as Effective KPC/AmpC Inhibitors

Joanna Krajewska [1], Piotr Chyży [2], Krzysztof Durka [3], Patrycja Wińska [3], Krystiana A. Krzyśko [4,*], Sergiusz Luliński [3] and Agnieszka E. Laudy [1,*]

[1] Department of Pharmaceutical Microbiology and Bioanalysis, Medical University of Warsaw, 02-097 Warsaw, Poland; joanna.krajewska@ymail.com (J.K.); alaudy@wp.pl A.E.L.)
[2] Centre of New Technologies, University of Warsaw, 02-097 Warsaw, Poland; p.chyzy@cent.uw.edu.pl (P.C.)
[3] Faculty of Chemistry, Warsaw University of Technology, 00-664 Warsaw, Poland; kdurka@gmail.com (K.D.); pwinska@ch.pw.edu.pl (P.W.); sergiusz.lulinski@pw.edu.pl (S.L.)
[4] Faculty of Physics, University of Warsaw, 02-093 Warsaw, Poland krystiana.krzysko@fuw.edu.pl (K.A.K.)
* Correspondence: krystiana.krzysko@fuw.edu.pl (K.A.K.); alaudy@wp.pl (A.E.L.)

List of contents

1. Synthetic procedures S2
1. NMR spectra of synthesized compounds S8
2. Antimicrobial activity S15
3. Cytotoxic activity S23
4. Molecular modeling and hybrid QM/MM simulations S24
5. References S31

1. Synthetic procedures

General comments. Solvents used for reactions were dried by heating to reflux with sodium/benzophenone and distilled under argon. Selected aromatic starting materials and other reagents including alkyllithiums, 2,2,6,6-tetramethylpiperidine, trialkyl borates, chlorotrimethylsilane, were used as received without further purification. In some cases, aromatic precursors were synthesized according to published procedures. In the ^{13}C NMR spectra the resonances of boron-bound carbon atoms were not observed in most cases as a result of their broadening by a quadrupolar boron nucleus. ^{1}H, and ^{13}C NMR chemical shifts are given relative to TMS using residual solvent resonances. ^{11}B and ^{19}F NMR chemical shifts are given relative to $BF_3{\cdot}Et_2O$ and $CFCl_3$, respectively.

*1.1. 2,5-Bis(trifluoromethyl)phenylene-1,4-diboronic acid (**1h**).*

A solution of 1,4-dibromo-bis(trifluoromethyl)benzene [94] (1.49 g; 4.0 mmol) in Et_2O (20 mL) was added to a solution of *t*-BuLi (1.9 M, 4.2 mL, 8 mmol) in THF (20 mL) at –90 °C. The mixture was stirred for 30 min at –100 °C followed by the dropwise addition of $B(OMe)_3$ (1.0 mL; 9.1 mmol). The resulting mixture was allowed to warm to ca. –50 °C followed by hydrolysis with aq. HCl (2 M, 5.0 mL). The organic phase was separated and the aqueous phase was extracted with Et_2O (30 mL). The combined organic phase was concentrated under reduced pressure and the solid residue was mixed with water (20 mL) and the resulting white suspension was filtered. The solid was washed with water (3 × 10 mL) and dichloromethane (10 mL), and dried under reduced pressure. The product **1h** was obtained as a white powder (0.95 g, yield 71%). ^{1}H NMR (400 MHz, DMSO-d_6) δ 8.54 (broad s, 4H), 7.77 (s, 2H) ppm. ^{13}C NMR (101 MHz, DMSO-d_6) δ 136.9 (broad), 133.0 (q, *J* = 30.5 Hz), 128.8 (q, *J* = 3.9 Hz), 124.4 (q, *J* = 274.1 Hz) ppm. ^{11}B NMR (96 MHz, DMSO-d_6) δ 29.0 ppm. ^{19}F NMR (376 MHz, DMSO-d_6) δ –59.01 ppm. HRMS (ESI, positive ion mode) calcd. for $C_8H_7B_2F_6O_4^+$ [MH^+]: 303.0429. Found: 303.0427.

*1.2. 2,5-Dibromophenylene-1,4-diboronic acid (**1i**).*

A solution of 2-(2,5-dibromophenyl)-6-butyl[1,3,6,2]dioxazaborocan (4.05 g, 10.0 mmol) in THF (50 mL) was cooled to –78 °C and $B(OiPr)_3$ (3.0 mL, 13.0 mmol) was added. A yellow solution of LTMP (obtained from 2,2,6,6-tetramethylpiperidine (2.2 mL, 13 mmol) and *n*-BuLi (2.5 M; 5.2 mL; 13.0 mmol) in THF (20 mL) at –78 °C) was added dropwise and the resulting mixture was stirred for 2 h at –78 °C. It was allowed to warm to 0 °C and hydrolyzed with aq. HCl (2 M, 40 mL). The organic phase was separated and the aqueous phase was extracted with Et_2O (50 mL). The combined organic phase was concentrated under reduced pressure and the solid residue was mixed with water (20 mL) and the resulting white suspension was washed with water (3 × 10 mL) and Et_2O (2 × 10 mL), and dried under reduced pressure. The product **1i** was obtained as a white powder (1.36 g, yield 42%). ^1H NMR (400 MHz, DMSO-d_6) δ 8.42 (s, 4H), 7.42 (s, 2H) ppm. $^{13}C\{^1H\}$ NMR (101 MHz, DMSO-d_6) δ 142.5 (broad), 136.4, 123.9 ppm. HRMS (ESI, positive ion mode) calcd. for $C_6H_7B_2Br_2O_4^+$ [MH^+]: 322.8892. Found: 322.8889.

*1.3. 5-Fluorophenylene-1,3-diboronic acid (**2b**).*

A solution of 1,3-dibromo-5-fluorobenzene (6.3 mL; 50 mmol) in Et_2O (15 mL) was added to a solution of *t*-BuLi (1.9 M, 105.3 mL, 200 mmol) in THF (300 mL) at –95 °C. It was stirred for 1 h at –90 °C followed by the dropwise addition of $B(OEt)_3$ (34 mL; 200 mmol). The resulting mixture was allowed to warm to ca. –80 °C followed by the addition of Me_3SiCl (27.9 mL; 200 mmol). The mixture was allowed to warm to the room temperature warm to the room temperature and left overnight with stirring. A mixture was concentrated under reduced pressure and hexane (50 mL) was added. The resulting suspension was filtered under argon and concentrated under reduced pressure. The residue was subjected to fractional distillation under reduced pressure: the main fraction was collected, b.p. 97–100 °C (p = 0.01 mbar). 5-Fluorophenylene-1,3-diboronic acid bis(diethyl ester) (**pre-2b**) was obtained as a colorless oil (11.0 g, yield: 84%). ^1H NMR (400 MHz, $CDCl_3$) δ 7.57 (d, J = 1.1 Hz, 1H), 7.28 (dd, J = 9.4, 1.0 Hz, 2H), 4.06 (q, J = 7.1 Hz, 8H), 1.26 (t, J = 7.0 Hz, 12H) ppm. ^{11}B NMR (96 MHz, $CDCl_3$) δ 27.8

ppm. ^{13}C NMR (101 MHz, $CDCl_3$) δ 162.2 (d, *J* = 247.0 Hz), 133.3 (d, *J* = 3.0 Hz), 120.7 (d, *J* = 19.4 Hz), 60.3, 17.4 ppm. ^{19}F NMR (376 MHz, $CDCl_3$) δ –114.98 ppm.

Compound **pre-2b** (4.55 g, mmol) was mixed with H_2O (10 mL): a white precipitate was formed immediately. It was filtered and dried under high vacuum (*p* = 0.01 mbar) to give the product **2b** (3.8 g, 95%). ^{1}H NMR (400 MHz, acetone-d_6) δ 8.06 (d, *J* = 1.2 Hz, 1H), 7.52 (dd, *J* = 9.7, 1.2 Hz, 2H), 4.04 (s, 4H) ppm. ^{13}C{^{1}H} NMR (101 MHz, acetone-d_6) δ 162.3 (d, *J* = 243.8 Hz), 135.8 (d, *J* = 2.5 Hz), 121.9 (d, *J* = 19.0 Hz). ^{11}B NMR (96 MHz, acetone-d_6) δ 28.5 ppm. ^{19}F NMR (376 MHz, acetone-d_6) δ –117.72 (t, *J* = 9.7 Hz) ppm. HRMS (ESI, positive ion mode) calcd. for $C_6H_8B_2FO_4^+$ [MH^+]: 185.0587. Found: 185.0588.

*1.4. 4,6-Difluorophenylene-1,3-diboronic acid (**2c**).*

A solution of 1,3-dibromo-4,6-fluorobenzene (5.44 g; 20 mmol) in Et_2O (30 mL) was added to a solution of *t*-BuLi (1.9 M, 42 mL, 80 mmol) in THF (100 mL) at –100 °C. The mixture was stirred for 30 min at –100 °C followed by the dropwise addition of $B(OMe)_3$ (8.8 mL; 80 mmol). The resulting mixture was allowed to warm to ca. –70 °C followed by hydrolysis with aq. HCl (2 M, 40 mL). The organic phase was separated and the aqueous phase was extracted with Et_2O (50 mL). The combined organic phase was concentrated under reduced pressure and the solid residue was mixed with water (20 mL) and the resulting white suspension was washed with water (3 × 10 mL) and Et_2O (10 mL), and dried under reduced pressure. The product **2c** was obtained as a white powder (2.86 g, yield 71%). ^{1}H NMR (400 MHz, DMSO- d_6) δ 8.13 (broad, 4H), 7.86 (t, *J* = 7.9 Hz, 1H), 6.94 (t, *J* = 9.8 Hz, 1H). ^{13}C{^{1}H} NMR (101 MHz, DMSO-d_6) δ 167.5 (dd, *J* = 250.3, 12.9 Hz), 143.2 (t, *J* = 11.2 Hz), 117.5, 102.7 (t, *J* = 27.9 Hz) ppm. ^{11}B NMR (96 MHz, DMSO-d_6) δ 29.0 ppm. ^{19}F NMR (376 MHz, DMSO-d_6) δ –98.66 (t, *J* = 9.0 Hz) ppm. HRMS (ESI, positive ion mode) calcd. for $C_6H_7B_2F_2O_4^+$ [MH^+]: 203.0493. Found: 203.0490.

*1.5. 4-(Trifluoromethyl)phenylene-1,2-diboronic acid (**3e**).*

2,2,6,6-Tetramethylpiperidine (17 mL, 0.10 mol) was added to a solution of *n*BuLi (11 M; 9.1 ml; 100 mmol) in THF (200 mL) at –78 °C. A yellow solution of LTMP was stirred for 30 min followed by the addition of $B(OiPr)_3$ (0.12 mol; 27.5 mL) at –78 °C. A solution of 1-bromo-4-(trifluoromethyl)benzene (100 mmol; 14 mL) in THF (50 mL) was added dropwise and the obtained green mixture was stirred for 1 h followed by addition of Me_3SiCl (16 mL; 120 mmol). The resulting pale yellow solution was allowed to warm to the room temperature and left overnight with stirring. A mixture was concentrated under reduced pressure and hexane (100 mL) was added. The resulting suspension was filtered under argon and concentrated under reduced pressure. The residue was subjected to fractional distillation under reduced pressure: the main fraction was collected, b.p. 84–86 °C (p = 0.01 mbar). 2-Bromo-5-trifluoromethyl-phenylboronic acid diisopropyl ester (**pre_I-3e**) was obtained as a colorless oil (21.2 g, yield: 60%). ^{1}H NMR (400 MHz, $CDCl_3$) δ 7.65–7.58 (m, 1H), 7.50–7.47 (m, 1H), 7.45 – 7.39 (m, 1H), 4.36 (hept, J = 6.1 Hz, 3H), 1.22 (d, J = 6.2 Hz, 20H) ppm. ^{19}F NMR (376 MHz, $CDCl_3$) δ –62.75 ppm. ^{11}B NMR (128 MHz, $CDCl_3$) δ 27.2 ppm. ^{13}C{^{1}H} NMR (101 MHz, $CDCl_3$) δ 132.0, 129.3 (q, J = 1.6 Hz), 129.04 (q, J = 32.6 Hz), 129.00 (q, J = 3.7 Hz), 126.6 (q, J = 3.6 Hz), 124.00 (q, J = 272.3 Hz), 67.2, 24.3 ppm.

Compound **pre_I-3e** (28.3 mmol; 10 g) was dissolved in hexane (70 mL) followed by the addition of *N*-butyldiethanolamine (28.3 mmol; 4.8 mL) diluted with hexane (20 mL). A white suspension was formed rapidly; it was left overnight with stirring and filtered under argon. The solid was washed with hexane (20 mL) and dried under reduced pressure to give 2-(2-bromo-5-(trifluoromethyl)phenyl)-6-butyl[1,3,6,2]dioxazaborocan (**pre_II-3e**) as a white solid (8.7 g, 78%). ^{1}H NMR (400 MHz, $CDCl_3$) δ 8.12–8.05 (m, 1H), 7.61 (dd, J = 8.3, 0.9 Hz, 1H), 7.33 (ddd, J = 8.3, 2.6, 0.7 Hz, 1H), 4.32–4.06 (m, 4H), 3.46–3.26 (m, 2H), 3.17–3.02 (m, 2H), 2.69–2.51 (m, 2H), 1.71–1.50 (m, 2H), 1.27–1.13 (m, 2H), 0.86 (t, J = 7.3 Hz, 3H) ppm. ^{11}B NMR (128 MHz, $CDCl_3$) δ 11.9 ppm. ^{19}F NMR (376 MHz, $CDCl_3$) δ –62.31 ppm.

A solution of **pre_II-3e** (1.97 g, 5.0 mmol) in THF (10 mL) was added to a solution of *n*BuLi (2.5 M; 2.2 mL; 5.5 mmol) in THF (30 mL) at –90 °C. The solution was stirred

for 1 h followed by addition of $B(OMe)_3$ (0.6 mL, 5.5 mmol). The resulting mixture was allowed to warm to ca. –70 °C followed by hydrolysis with aq. HCl (2 M, 10 mL). The organic phase was separated and the aqueous phase was extracted with Et_2O (50 mL). The combined organic phase was concentrated under reduced pressure and the solid residue was mixed with water (5 mL) and the resulting white suspension was washed with water (2 × 5 mL), and dried under reduced pressure. The product **2c** was obtained as a white powder (1.03 g, yield 90%). ^{1}H NMR (400 MHz, acetone-d_6) δ 7.79–7.72 (m, 2H), 7.58 (ddd, J = 8.5, 2.5, 0.8 Hz, 1H) ppm. ^{13}C{^{1}H} NMR (101 MHz, acetone-d_6) δ 132.8, 130.7 (q, J = 3.8 Hz), 130.1–129.7 (m), 128.2 (q, J = 32.3 Hz), 127.0 (q, J = 3.8 Hz), 124.4 (q, J = 271.3 Hz) ppm. ^{11}B NMR (128 MHz, acetone-d_6) δ 28.7 ppm. ^{19}F NMR (376 MHz, acetone-d_6) δ –63.13 ppm. HRMS (ESI, positive ion mode) calcd. for $C_7H_8B_2F_3O_4^+$ [MH^+]: 235.0555. Found: 235.0557.

*1.6. Pyridine-3,5-diboronic acid hydrochloride (**6a**).*

n-BuLi (10 M in hexane, 5.0 mL, 0.05 mol) was added to the suspension of 3,5-dibromopyridine (12.9 g, 0.05 mol) in Et_2O (100 mL) at –78 °C. The mixture was stirred for 2 h and B(O*i*Pr)$_3$ (11.5 mL, 0.05 mol) was added dropwise. The mixture was stirred for 1 h and THF (100 mL) was added followed by dropwise addition of *t*-BuLi (1.9 M in pentane, 53 mL, 0.10 mol) at –78 °C. The mixture was stirred for 30 min and B(O*i*Pr)$_3$ (16.1 mL, 0.07 mol) was added dropwise. The mixture was allowed to warm to –20 °C and quenched with Me_3SiCl (25 mL, 0.20 mol). The mixture was allowed to warm to room temperature and left overnight with stirring. The resulting suspension was filtered under argon and the solid was washed with Et_2O (2 × 30 mL). The collected filtrate was concentrated under reduced pressure. The residue was subjected to fractional distillation under reduced pressure: the main fraction was collected, b.p. 145–150 °C (p = 0.01 mbar). The crude pyridine-3,5-diboronic acid bis(diisopropyl ester) (**pre-6a**) distilled as a pale yellow oil rapidly solidifying upon cooling (13.6 g, yield: 81%).

Compound **pre-6a** (3.35 g, 10.0 mmol) was dissolved in acetone (20 mL) and H_2O (0.81 g, 45 mmol) was added dropwise. The obtained white suspension was acidified

with HCl (2 M in Et_2O, 5.0 mL, 10.0 mmol) and the mixture was evaporated under reduced pressure. The solid residue was triturated with Et_2O (20 mL). The white suspension was stirred for 1 h and filtered. The solid was washed with Et_2O (10 mL), dichloromethane (10 mL) and dried to give **6a** as a white powder (1.13 g, 68%). ^1H NMR (400 MHz, DMSO-d6) δ 9.12 (s, 1H), 9.02 (d, J = 1.6 Hz, 3H) ppm. 13C{1H} NMR (101 MHz, DMSO-d6) δ 156.8, 146.3, 132.4 (broad) ppm. HRMS (ESI, positive ion mode) calcd. for C5H8B2NO4+ [MH+]: 168.0634. Found: 168.0635.

*1.7. 2-Mercaptophenylboronic acid (**7c**).*

A solution of 2-bromothiophenol (1.89 g, 10.0 mmol) in THF (10 mL) was added dropwise to a suspension of NaH (60wt% dispersion in mineral oil, 0.50 g, 12 mmol, washed with hexane prior to use) in THF (20 mL) at 0 °C. The mixture was stirred for 1 h and cooled to −78 °C followed by addition of a solution of *t*-BuLi (1.9 M, 10.5 mL, 20.0 mmol) at −78 °C. The mixture was stirred for 30 min followed by the dropwise addition of $B(OMe)_3$ (2.2 mL; 20.0 mmol). The resulting mixture was allowed to warm to ca. −50 °C followed by hydrolysis with aq. HCl (2 M, 5.0 mL). The organic phase was separated and the aqueous phase was extracted with Et_2O (30 mL). The combined organic phase was concentrated under reduced pressure and the solid residue was mixed with water (5 mL) and the resulting white suspension was filtered. The solid was washed with water (3 × 3 mL) and hexane (5 mL), and dried under reduced pressure. The product **7c** was obtained as a white powder (0.74 g, yield 48%). ^1H NMR (400 MHz, acetone-d_6) δ 7.83 (dd, *J* = 7.3, 1.6 Hz, 1H), 7.80 (s, 1H), 7.38 (td, *J* = 7.6, 1.6 Hz, 1H), 7.21–7.16 (m, 2H) ppm. $^{13}C\{^1H\}$ NMR (101 MHz, acetone-d_6) δ 142.9, 135.1, 132.6, 127.8, 125.8 ppm. ^{11}B NMR (96 MHz, acetone-d_6) δ 27.2 ppm. HRMS (ESI, positive ion mode) calcd. for $C_6H_8BO_2S^+$ [MH^+]: 155.0333. Found: 155.0334.

1. NMR spectra of synthesized compounds

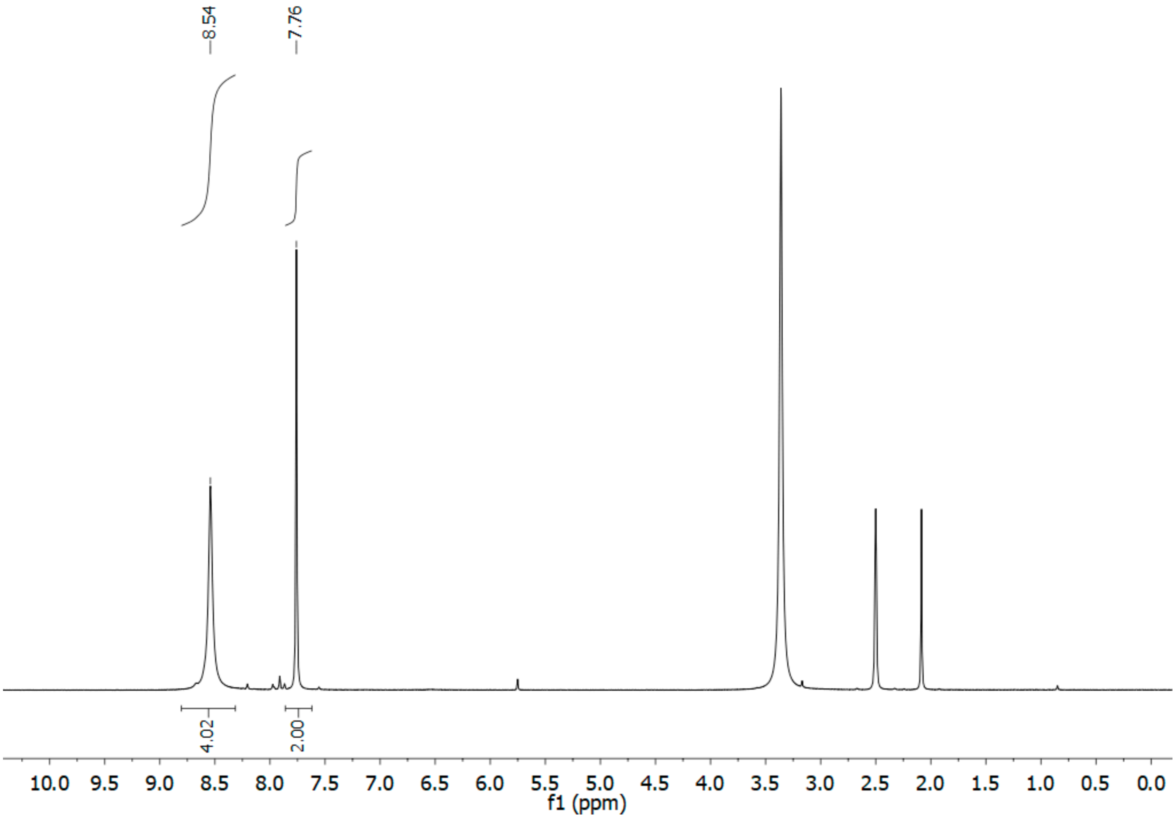

Figure S1. ^{1}H NMR (400 MHz, DMSO-d_6) spectrum of **1h**.

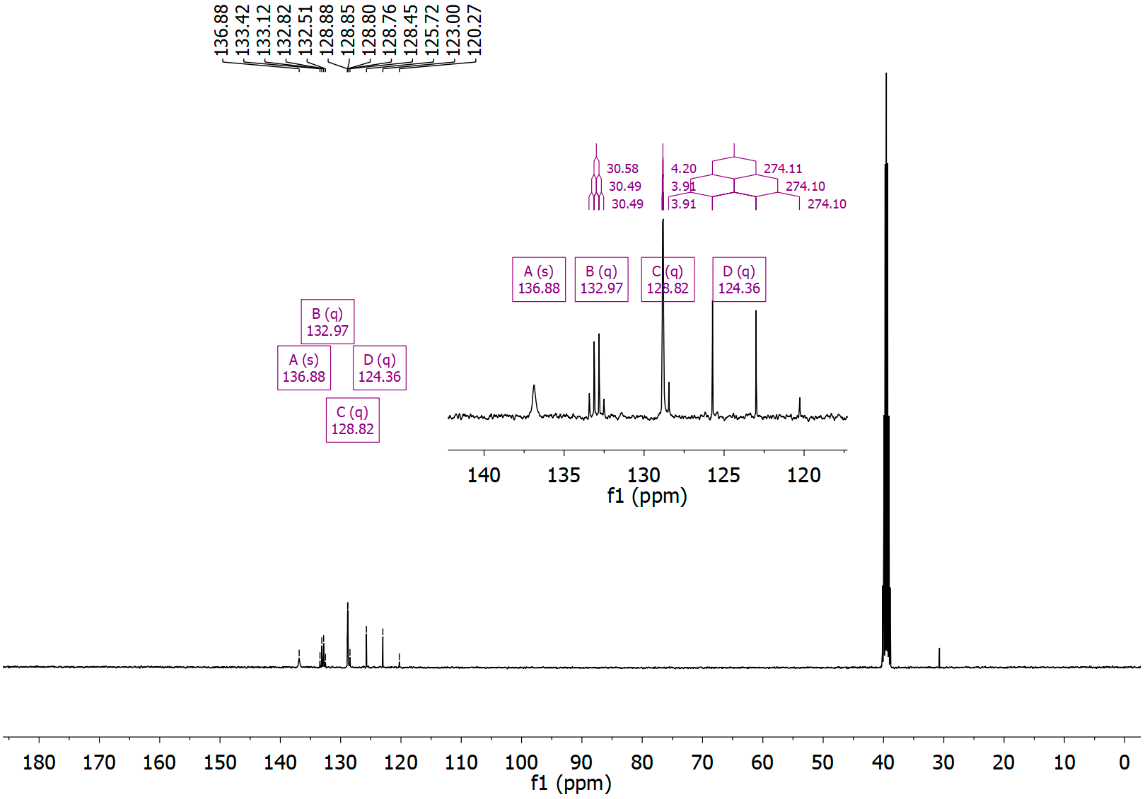

Figure S2. $^{13}C\{^{1}H\}$ NMR (400 MHz, DMSO-d_6) spectrum of **1h**.

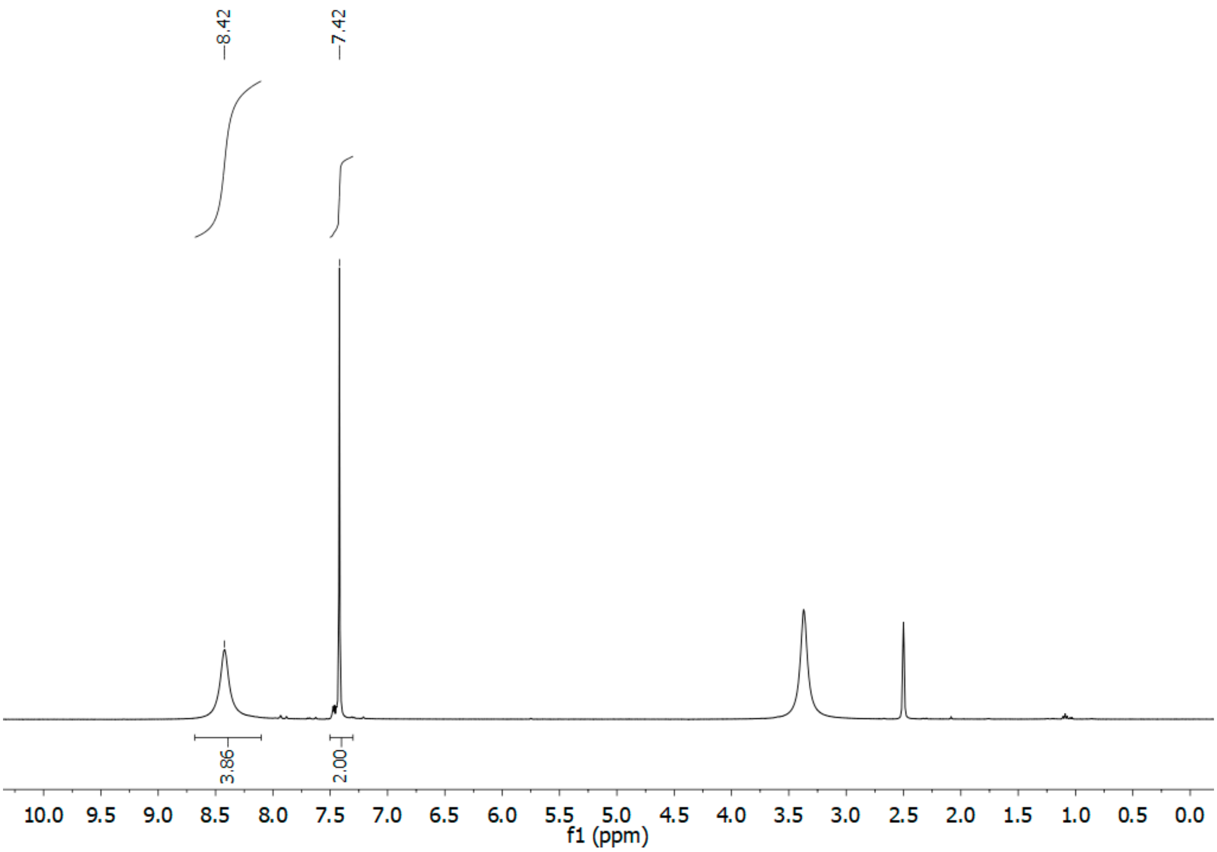

Figure S3. ^{1}H NMR (400 MHz, DMSO-d_6) spectrum of **1i**.

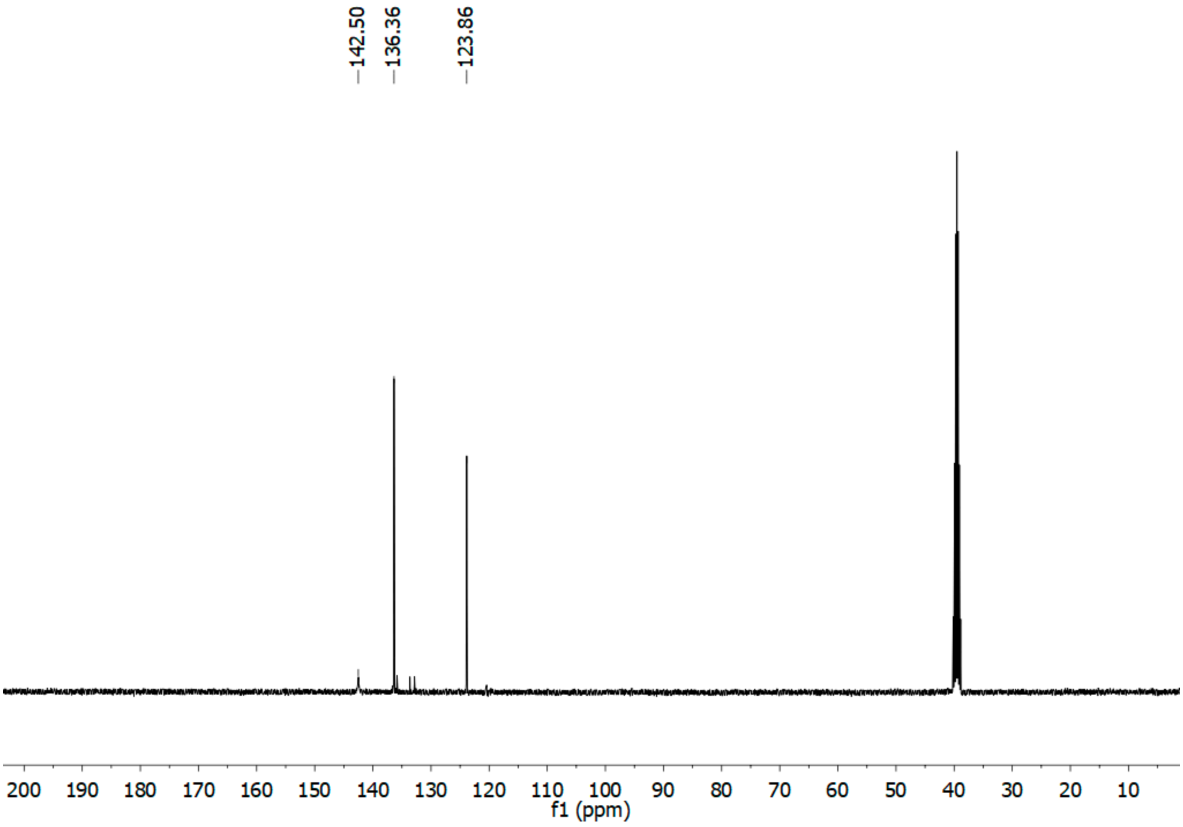

Figure S4. ^{13}C{^{1}H} NMR (100 MHz, DMSO-d_6) spectrum of **1i**.

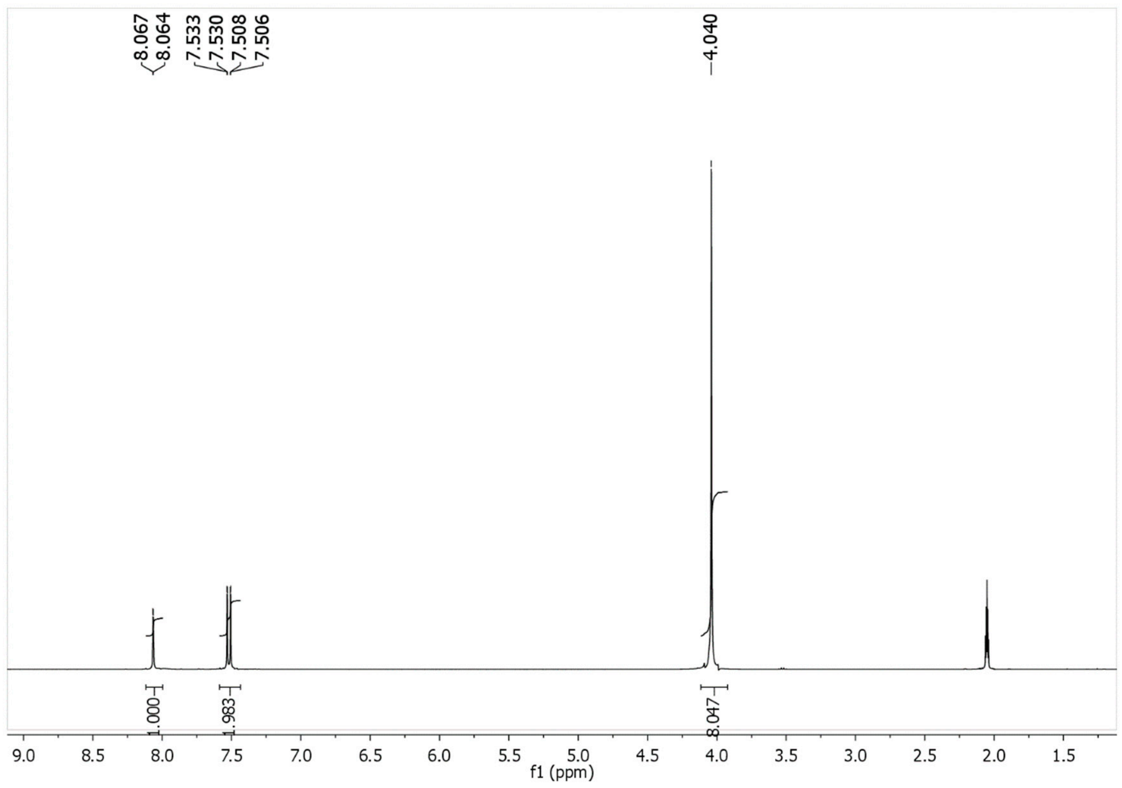

Figure S5. ^{1}H NMR (400 MHz, acetone-d_6) spectrum of **2b**.

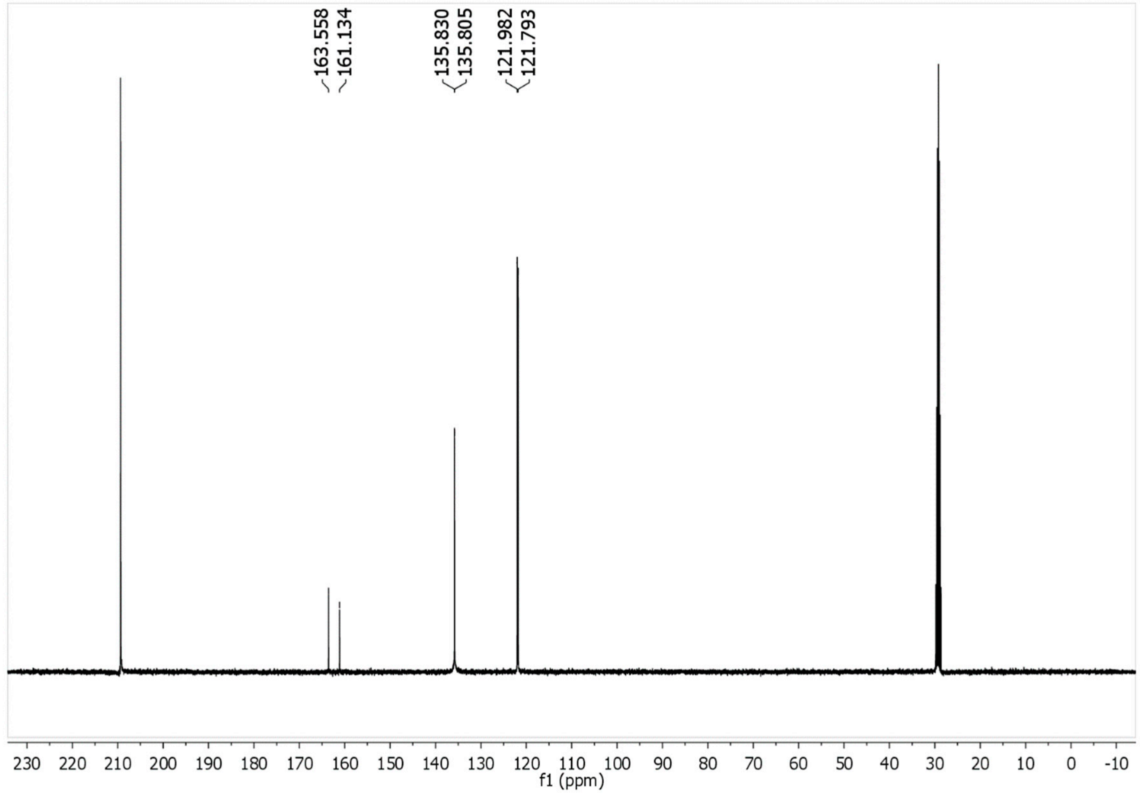

Figure S6. ^{13}C{^{1}H} NMR (100 MHz, acetone-d_6) spectrum of **2b**.

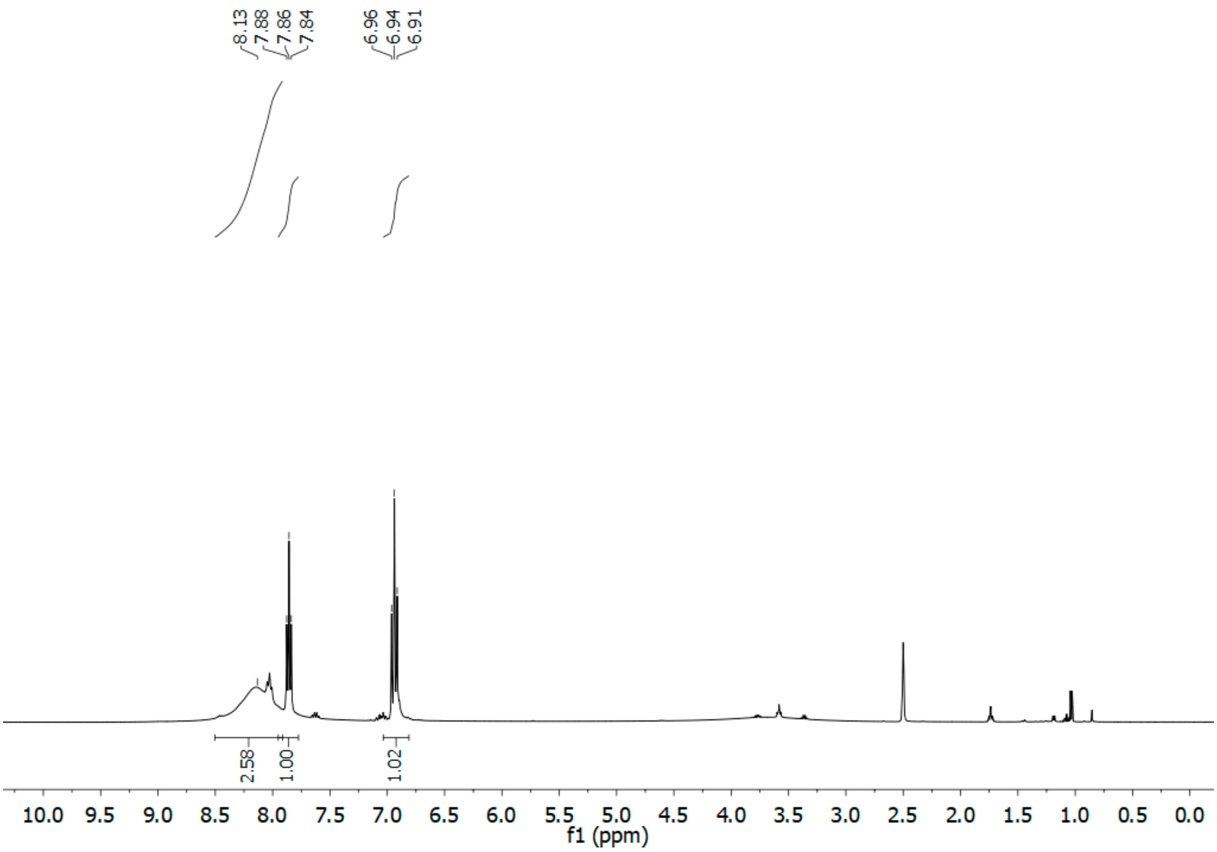

Figure S7. ^{1}H NMR (400 MHz, DMSO-d_6) spectrum of **2c**.

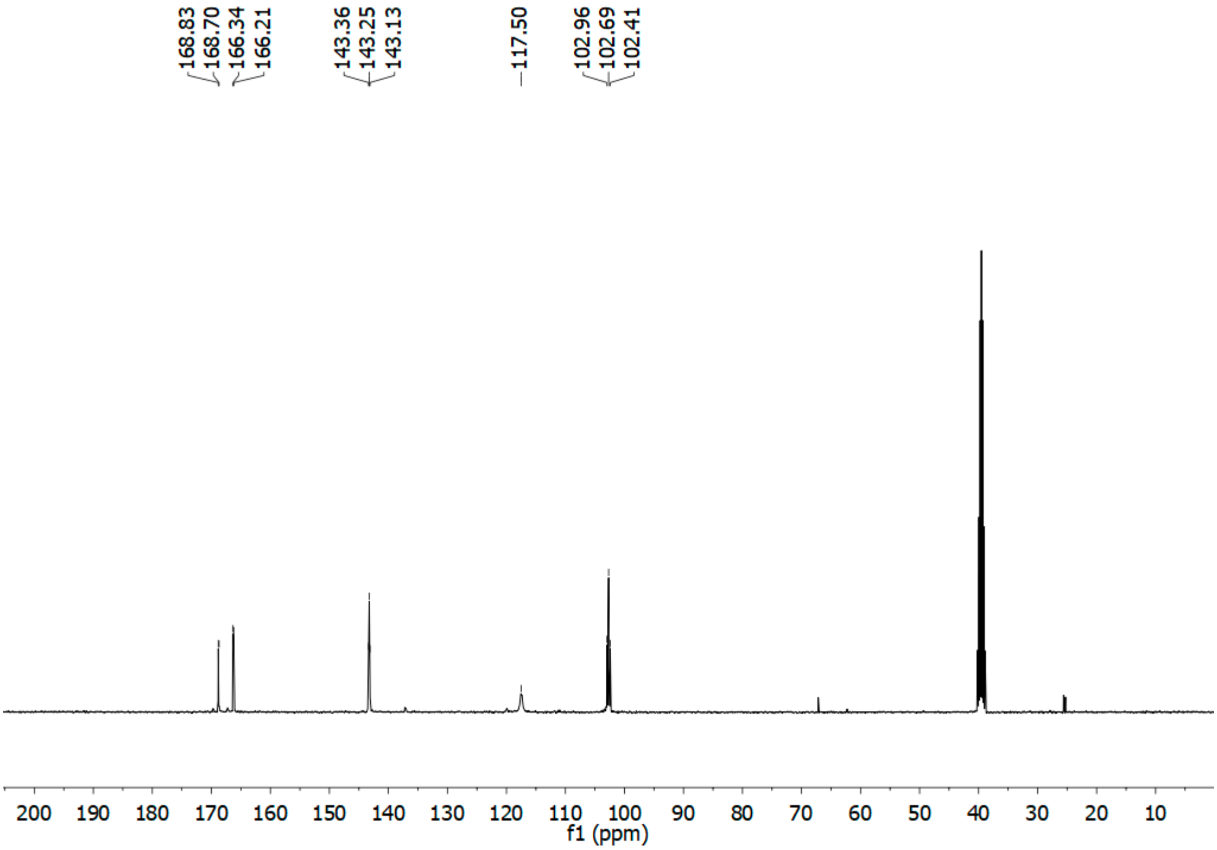

Figure S8. ^{13}C{^{1}H} NMR (100 MHz, DMSO-d_6) spectrum of **2c**.

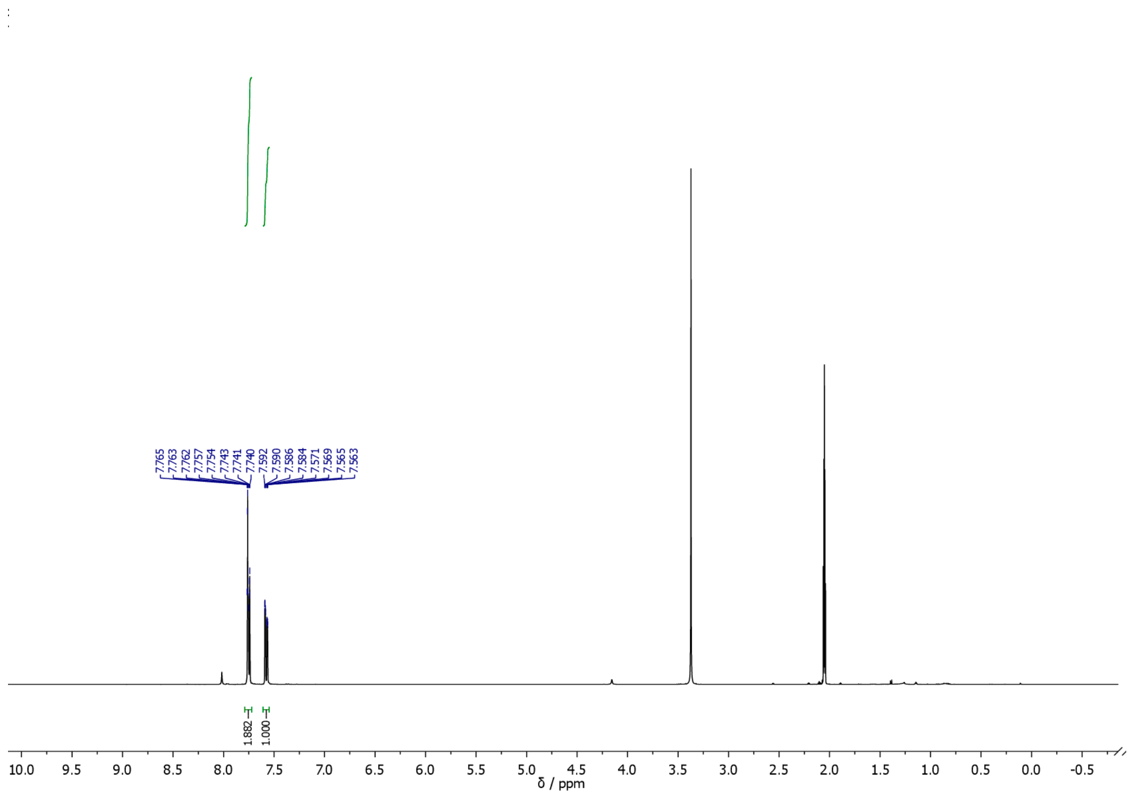

Figure S9. ^{1}H NMR (400 MHz, acetone-d_6) spectrum of **3e**.

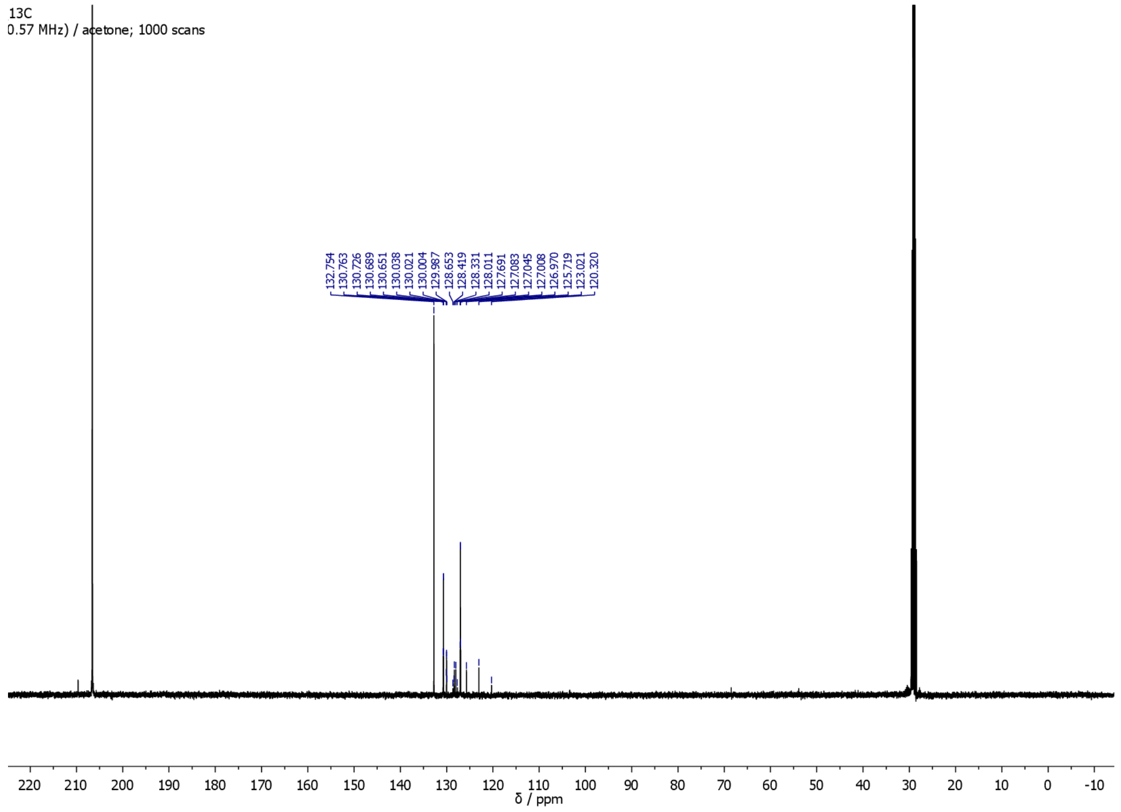

Figure S10. $^{13}C\{^{1}H\}$ NMR (100 MHz, acetone-d_6) spectrum of **3e**.

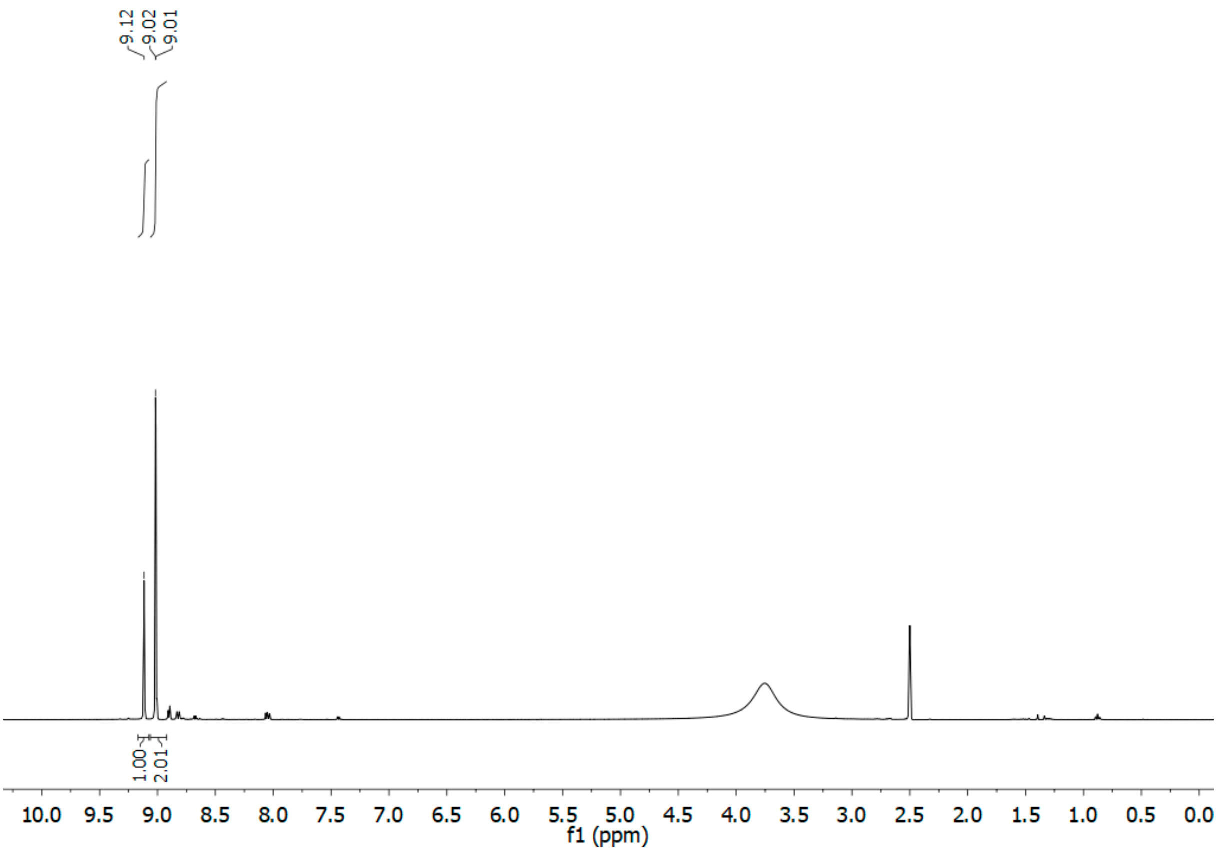

Figure S11. ^{1}H NMR (400 MHz, DMSO-d_6) spectrum of **6a**.

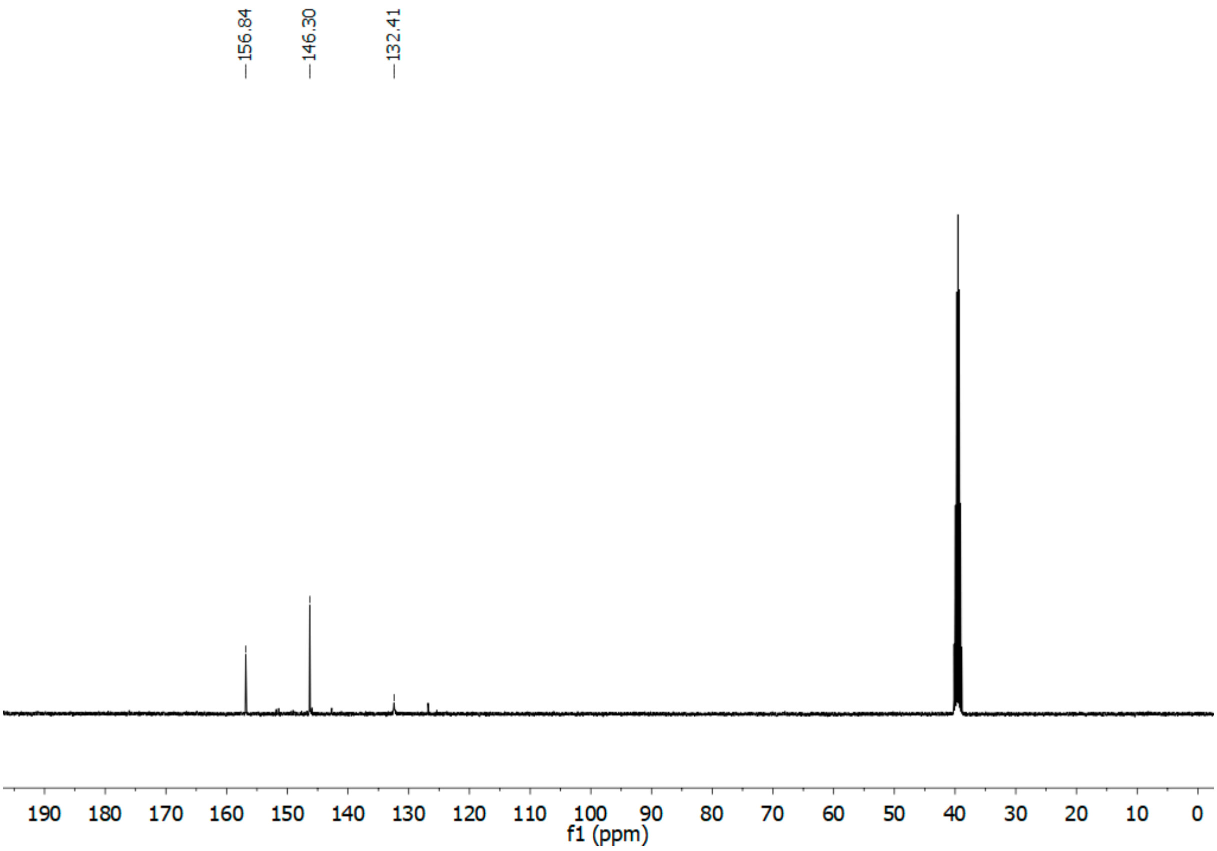

Figure S12. $^{13}C\{^{1}H\}$ NMR (100 MHz, DMSO-d_6) spectrum of **6a**.

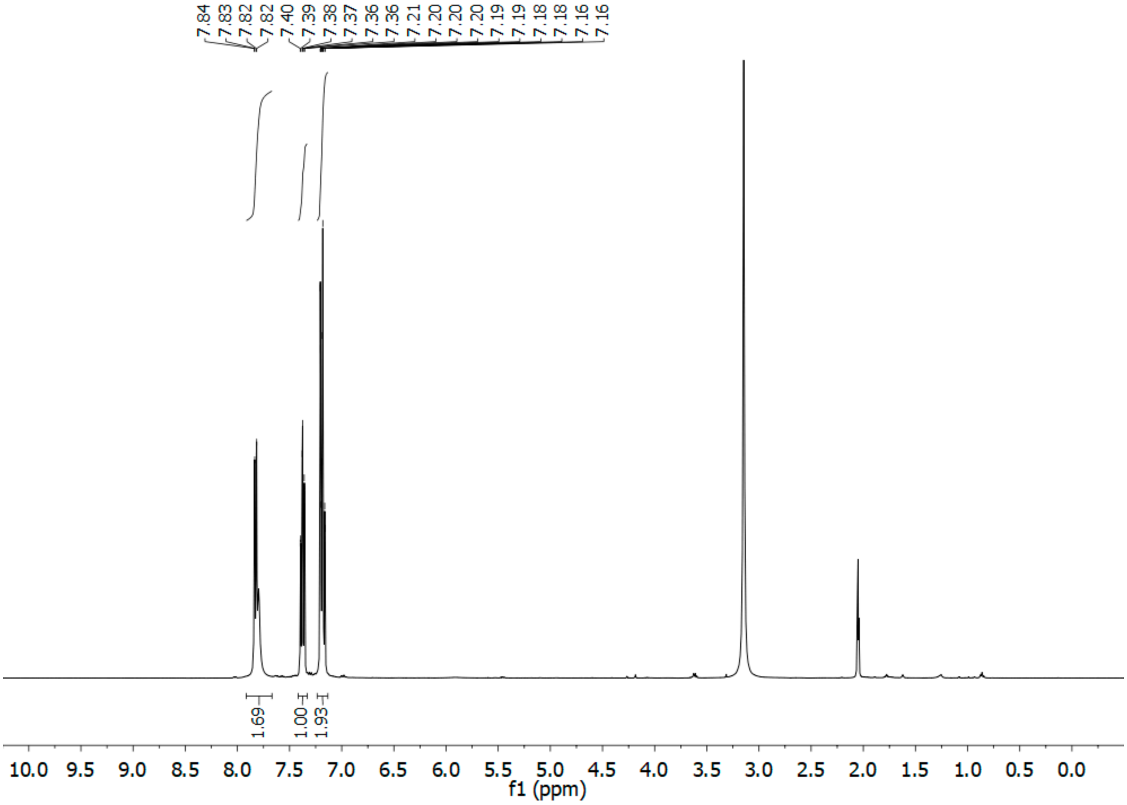

Figure S13. ^{1}H NMR (400 MHz, acetone-d_6) spectrum of **7c**.

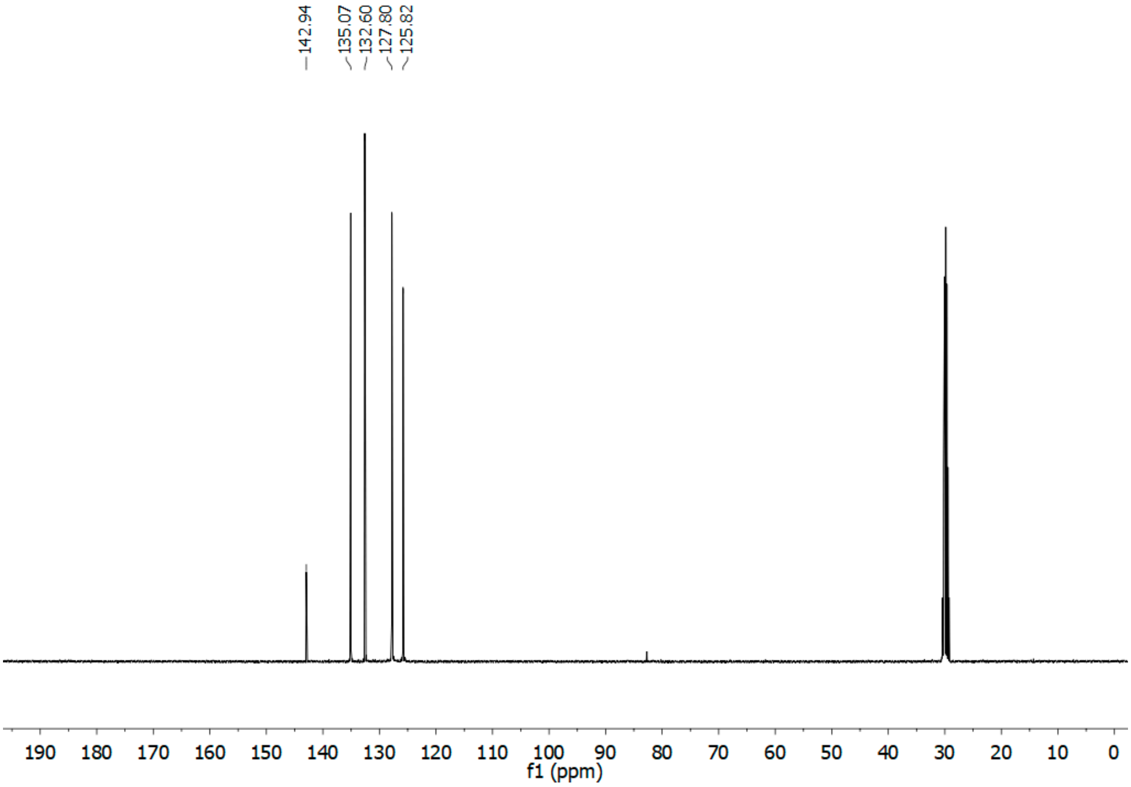

Figure S14. $^{13}C\{^{1}H\}$ NMR (100 MHz, acetone-d_6) spectrum of **7c**.

2. Antimicrobial activity

Table S1. The antibacterial activity of tested agents against standard Gram-positive strains.

Agent tested	MIC in mg/L [MBC in mg/L][a] (Diameter of inhibition zone in mm) *S. aureus* ATCC 6538P	*S. aureus* ATCC 43300 MRSA	*S. epidermidis* ATCC 12228	*E. faecalis* ATCC 29212	*E. faecium* ATCC 6057	*B. subtilis* ATCC 6633[b]
**1a**	200 (-)	400 (-)	400 (14)	200 (-)	400 (-)	NT (17)
**1b**	25 (14)	100 (13)	100 (17)	200 (-)	200 (-)	NT (23)
**1c**	25 (22)	50 (22)	50 (28)	200 (16)	200 (15)	NT (28)
**1d**	25 (-)	200 (-)	25 (21)	>400 (-)	>400 (-)	NT (15)
**1e**	**12.5** (20)	25 (26)	25 (21)	200 (15)	100 (14)	NT (28)
**1f**	50 (17)	200 (16)	50 (17)	400 (-)	200 (-)	NT (13)
**1g**	>400 (-)	>400 (-)	>400 (-)	>400 (-)	>400 (-)	NT (-)
**1h**	400 (13)	400 (14)	200 (-)	400 (-)	400 (-)	NT (12)
**1i**	25 (14)	100 (11)	50 (21)	200 (14)	200 (11)	NT (18)
**1j**	50 (19)	50 (21)	50 (25)	400 (-)	400 (-)	NT (20)
**1k**[c]	100 (-)	100 (-)	100 (-)	>100 (-)	100 (-)	NT (11)
**1l**	NT (-)	NT (-)	NT (-)	NT (-)	NT (-)	NT (-)
**2a**	100 (20)	400 (-)	200 (21)	200 (-)	>400 (-)	NT (18)
**2b**	100 (14)	400 (15)	50 (19)	400 (-)	400 (14)	NT (17)
**2c**	50 (20)	100 (19)	50 (-)	400 (-)	400 (-)	NT (-)
**2d**	50 (22)	100 (19)	50 (12)	200 (14)	200 (14)	NT (15)
**2e**	**6.25** (29)	**12.5** (25)	**0.78** (35)	50 (21)	100 (18)	NT (20)
**2f**	50 [400] (14)	200 (13)	100 (25)	>400 (-)	200 (-)	NT (22)
**2g**	>400 (-)	>400 (-)	>400 (-)	>400 (-)	400 (-)	NT (-)
**3a**	200 (-)	400 (-)	200 (20)	400 (-)	50 (16)	NT (24)
**3b**	>400 (-)	>400 (-)	400 (21)	>400 (-)	>400 (-)	NT (-)
**3c**	200 (13)	400 (11)	100 (24)	400 (-)	400 (12)	NT (18)
**3d**	200 (20)	400 (18)	200 (21)	200 (12)	200 (16)	NT (12)
**3e**	100 (17)	400 (15)	200 (21)	200 (14)	200 (13)	NT (22)
**4a**	>400 (-)	>400 (-)	>400 (-)	>400 (-)	400 (-)	NT (-)
**4b**	25 (20)	200 (-)	200 (20)	200 (-)	200 (-)	NT (18)
**5a**	100 (-)	400 (-)	400 (13)	100 (-)	>400 (-)	NT (15)
**5b**	>400 (-)	>400 (-)	>400 (-)	200 (-)	>400 (-)	NT (-)
**5c**	400 (-)	>400 (-)	>400 (-)	200 (-)	>400 (-)	NT (-)
**6a**	50 (-)	400 (-)	400 (-)	>400 (-)	400 (-)	NT (-)
**7a**	50 (-)	100 (-)	100 (-)	400 (-)	400 (-)	NT (-)
**7b**	50 [100] (19)	50 (20)	50 [100] (30)	200 (14)	200 (14)	NT (22)
**7c**[d]	**12.5** (23)	25 (22)	**12.5** (30)	25 (23)	**12.5** (25)	NT (26)
LIN[e]	1 [>128] (25)	2 [>128] (25)	1 [>128] (26)	2 [>128] (15)	2 [>128] (14)	NT (30)

The highest activity against Gram-positive bacteria indicated by the low MIC values (≤**12.5** mg/L) is shown in boldface.

(-) – The inhibition zone was not observed in the disc-diffusion method. The diameter of paper discs was 9 mm; NT – not tested.

[a] Only the MBC values ≤400 mg/L are presented.

[b] The growth type of *B. subtilis* in the MHB medium prevented reading the MIC values of tested substances.

[c] The MIC and MBC values of the substance were determined up to 100 mg/L. In the table, only the MBC values ≤100 mg/L are presented. The tested substance dissolved in DMSO precipitated after implementation into the MHB medium at a concentration above 100 mg/L.

[d] The MIC and MBC values of the substance were determined up to 200 mg/L. In the table, only the MBC values ≤200 mg/L are presented. The tested substance dissolved in DMSO precipitated after implementation into the MHB medium at a concentration above 200 mg/L.

[e] LIN, linezolid was used as a reference agent active against Gram-positive bacteria. The diameter of the commercial disc containing 0.03 mg of linezolid was 6 mm; the MIC of linezolid was determined according to the CLSI recommendations [75].

Table S2. The antibacterial activity of tested agents against standard Gram-negative strains.

Agent tested	MIC in mg/L [MBC in mg/L][a] / x-fold reduction of MIC in the presence of PAßN[b] (Diameter of inhibition zone in mm)									
	E. coli **ATCC 25922**	***K. pneumoniae*** **ATCC 13883**	***P. mirabilis*** **ATCC 12453**	***E. cloacae*** **DSM 6234**	***S. marcescens*** **ATCC 13880**	***P. aeruginosa*** **ATCC 27853**	***S. maltophilia*** **ATCC 13637**	***A. baumannii*** **ATCC 19606**	***B. cepacia*** **ATCC 25416[c]**	***B. bronchiseptica*** **ATCC 4617[c]**
**1a**	>400 (-)	>400 (-)	>400 (-)	>400 (-)	>400 (-)	>400 (-)	>400 (-)	>400 (-)	>400 (11)	400 [400] (13)
**1b**	400 (19)	400 (-)	400/2 (17)	400/2 (-)	400/2 (-)	>400/≥2 (-)	400/2 (18)	400 (-)	400 (15)	100 (21)
**1c**	200 (20)	200 (-)	200 (16)	200 (-)	200/2 (-)	400/2 (-)	100 (21)	400/2 (12)	200 (20)	50 (24)
**1d**	200 (-)	400 (-)	200 (-)	200 (-)	400 (18)	>400 (-)	200 (12)	200 (-)	400 (14)	100 (15)
**1e**	100 [200] (-)	200 [400]/2 (16)	100 (-)	100 (18)	100 (18)	400/2 (-)	50 [400] (20)	200/2 (14)	200 (18)	50 [200] (23)
**1f**	100 [100] (17)	100 [100] (21)	100 (17)	100 [100] (17)	100 (21)	>400 (-)	200/2 (14)	100 (16)	50 (20)	50 (20)
**1g**	>400 (-)	>400 (-)	>400 (-)	>400 (-)	>400 (-)	>400 (-)	>400 (-)	>400 (-)	>400 (-)	>400 (-)
**1h**	>400/≥2 (-)	>400 (-)	>400 (-)	>400 (-)	>400 (-)	>400 (-)	>400/≥2 (-)	>400 (-)	>400 (-)	>400 (-)
**1i**	100/**4** (17)	200/2 (13)	100/2 (21)	100/2 (19)	200/**4** (21)	>400/≥2 (-)	50 (17)	100/2 (17)	400 (16)	100 (14)
**1j**	100/4 (14)	400/2 (14)	200/2 (17)	200/2 (17)	200/2 (18)	>400 (-)	50 [400](15)	50 (15)	200 (17)	100 (20)
**1k[d]**	>100 (-)	>100 (-)	>100 (-)	>100 (-)	>100 (-)	>100 (-)	>100 (-)	>100 (-)	>100 (-)	>100 (-)
**1l**	NT (-)	NT (-)	NT (-)	NT (-)	NT (-)	NT (-)	NT (-)	NT (-)	NT (-)	NT (-)
**2a**	>400 (-)	>400 (-)	>400 (-)	>400 (-)	>400 (-)	>400 (-)	400 (-)	>400 (-)	>400 (15)	200 (12)
**2b**	400 (-)	400 (17)	400 (-)	400 (-)	400 (-)	>400 (-)	100 (19)	>400/≥2 (-)	400 (12)	100 (18)
**2c**	200 (12)	200 (12)	400 (12)	200 (12)	200 (11)	>400 (-)	50 (21)	400 (13)	200 (13)	50 (22)
**2d**	200 (12)	400 (12)	400 (11)	400 (11)	100 (11)	>400 (-)	50 (29)	400/2 (11)	400 (-)	50 (20)
**2e**	400/2 (-)	400/**4** (-)	>400/**≥4** (-)	>400/**≥4** (-)	400/2 (-)	>400/**≥4** (-)	25 (15)	400/2 (-)	400 (14)	100 (14)
**2f**	>400 (13)	>400 (11)	>400 (-)	>400/≥2 (-)	>400/≥2 (-)	>400 (-)	100 (18)	>400/≥2 (-)	>400 (-)	200 (13)
**2g**	>400 (-)	>400 (-)	>400 (-)	>400 (-)	>400 (-)	>400 (-)	>400 (-)	>400 (-)	>400 (-)	>400 (-)
**3a**	400/2 (-)	400 (-)	400 (-)	400/2 (-)	400 (-)	400/2 (-)	400/2 (-)	400 (-)	400 (-)	400 (-)
**3b**	>400 (-)	>400 (-)	>400 (-)	>400/≥2 (-)	>400 (-)	>400/≥2 (-)	>400/≥2 (13)	>400 (-)	>400 (-)	>400 (-)
**3c**	>400/≥2 (-)	>400/≥2 (-)	>400/≥2 (-)	>400/≥2 (-)	>400/≥2 (-)	>400/≥2 (11)	>400 (12)	>400 (-)	>400 (-)	400 (12)
**3d**	>400/≥2 (-)	>400/≥2 (-)	>400/≥2 (-)	>400/≥2 (-)	>400 (-)	>400/≥2 (11)	200/2 (12)	400/2 (-)	>400 (-)	>400 (-)
**3e**	>400/**≥8** (13)	>400/**≥4** (12)	>400/≥2 (13)	>400/**≥8** (-)	>400/**≥16** (9)	>400/**≥16** (-)	200 [400]/**4** (14)	400/**4** (-)	>400 (10)	400 (-)
**4a**	>400 (-)	>400 (-)	>400 (-)	>400 (-)	>400 (-)	>400 (-)	>400 (-)	>400 (-)	>400 (-)	>400 (-)
**4b**	>400 (-)	>400 (-)	>400 (-)	>400 (-)	>400 (-)	>400 (-)	>400/≥2 (-)	>400 (-)	>400 (11)	400 (-)
**5a**	>400 (-)	>400 (-)	>400 (-)	400 (-)	>400 (-)	>400/**≥4** (-)	400 (-)	>400 (-)	400 (-)	200 (20)
**5b**	>400 (-)	>400 (-)	>400 (-)	>400 (-)	>400 (-)	>400 (-)	>400 (-)	>400 (-)	>400 (-)	>400 (-)
**5c**	>400 (-)	>400 (-)	>400 (-)	>400 (-)	>400 (-)	>400 (-)	>400 (-)	>400 (-)	>400 (-)	400 (-)

**6a**	>400 (-)	>400 (-)	>400 (-)	>400 (-)	>400 (-)	>400 (-)	>400 (-)	>400/≥2 (-)	>400 (-)	>400 (-)
**7a**	50 (20)	100 (22)	100 (22)	100/2 (22)	50 (25)	400 (-)	100 (19)	100/2 (18)	50 (23)	50 (22)
**7b**	100/2 (18)	400/8 (13)	400/4 (11)	400/8 (14)	400/8 (15)	>400/≥16 (11)	12,5 [400]/4 (17)	100/2 (20)	400 (16)	50 (20)
**7c**[e]	>200/**≥8** (15)	>200/≥2 (-)	>200/**≥4** (-)	>200/**≥4** (-)	>200/≥2 (-)	>200 (-)	50 [100]/2 (15)	>200/**≥16** (13)	>200 (15)	50 (19)
Nf[f]	**8 [8] (24)**	**32 [32] (23)**	**128 [>128]**	**32 [32] (17)**	**128 [>128] (12)**	**>128 [>128] (-**	**128 [>128] (-)**	**64 [128] (9)**	**32 [32] (12)**	**64 [128] (-)**

The significant decreases (at least a 4-fold) in the MIC values of tested compounds after the addition of PAβN are shown in boldface. The test was performed in the MHB medium supplemented with 1 mM $MgSO_4$.
PAβN – efflux pump inhibitor; (-) – The inhibition zone was not observed in the disc-diffusion method. The diameter of the paper discs was 9 mm; NT – not tested. The MIC determination could not be performed, because the tested substance dissolved in DMSO precipitated after implementation into the MHB (Mueller-Hinton II broth) medium.
[a] Only the MBC values ≤400 mg/L are presented.
[b] In the table, only at least 2-fold decreases in the MIC values of tested compounds after the addition of PAβN are presented.
[c] The growth of *B. cepacia* ATCC 25416 and *B. bronchiseptica* ATCC 4617 strains was inhibited in the MHB medium supplemented with 1 mM $MgSO_4$ and 20 mg/L PAβN.
[d] The MIC and MBC values of the substance were determined up to 100 mg/L. In the table, only the MBC values ≤100 mg/L are presented. The tested substance dissolved in DMSO precipitated after implementation into the MHB medium at a concentration above 100 mg/L.
[e] The MIC and MBC values of the substance were determined up to 200 mg/L. In the table, only the MBC values ≤200 mg/L are presented. The tested substance dissolved in DMSO precipitated after implementation into the MHB medium at a concentration above 200 mg/L.
[f] Nf, nitrofurantoin was used as a reference agent active against Gram-negative bacteria. The diameter of a commercial disc containing 0.3 mg of nitrofurantoin was 6 mm; the MIC of nitrofurantoin was determined according to the CLSI recommendations [75].

Table S3. The antifungal activity of tested agents against yeast strains.

Agent tested	MIC in mg/L [MFC in mg/L][a] (Diameter of inhibition zone in mm) *C. albicans* ATCC 90028	*C. parapsilosis* ATCC 22019	*C. tropicalis* IBA 171	*C. tropicalis* ATCC 750	*C. guilliermondii* IBA 155	*C. krusei* ATCC 6258	*S. cerevisiae* ATCC 9763
**1a**	>400 (-)	>400 (-)	>400 (-)	>400 (-)	>400 (-)	>400 (-)	400 (-)
**1b**	>400 (-)	100 (12)	400 (-)	>400 (-)	400 (24)	100 (22)	25 (14)
**1c**	>400 (-)	>400 (-)	>400 (-)	>400 (-)	>400 (-)	400 (-)	200 (-)
**1d**	200 (-)	200 (14)	200 (-)	400 (-)	200 (16)	200 (-)	200 (13)
**1e**	>400 (-)	>400 (15)	>400 (-)	>400 (-)	>400 (16)	>400 (16)	400 (13)
**1f**	50 (24)	100 (20)	50 (20)	100 (18)	50 (29)	100 (19)	50 (20)
**1g**	>400 (-)	>400 (-)	>400 (-)	>400 (-)	>400 (-)	400 (-)	>400 (-)
**1h**	>400 (-)	>400 (-)	>400 (-)	>400 (-)	>400 (-)	>400 (-)	>400 (-)
**1i**	**12.5 (31)**	50 (24)	25 (29)	50 (25)	25 (34)	50 (22)	50 (24)
**1j**	200 (24)	>400 (15)	>400 (15)	>400 (17)	400 (30)	400 (23)	>400 (20)
**1k**[b]	>100 (-)	>100 (-)	>100 (-)	>100 (-)	>100 (-)	>100 (-)	>100 (-)
**1l**	NT (-)	NT (-)	NT (-)	NT (-)	NT (-)	NT (-)	NT (-)
**2a**	400 (-)	>400 (-)	400 (-)	>400 (-)	>400 (14)	>400 (-)	>400 (-)
**2b**	>400 (-)	>400 (-)	>400 (-)	>400 (-)	>400 (-)	>400 (-)	>400 (-)
**2c**	>400 (-)	>400 (-)	>400 (-)	>400 (-)	>400 (-)	>400 (-)	>400 (-)
**2d**	400 (-)	>400 (-)	>400 (-)	>400 (-)	400 (-)	400 (-)	>400 (-)
**2e**	400 (-)	400 (-)	400 (-)	>400 (-)	>400 (14)	>400 (-)	>400 (-)
**2f**	>400 (-)	>400 (-)	>400 (-)	>400 (-)	>400 (-)	400 (-)	200 (-)
**2g**	>400 (-)	>400 (-)	>400 (-)	>400 (-)	>400 (-)	>400 (-)	>400 (-)
**3a**	>400 (-)	25 (-)	>400 (-)	>400 (-)	>400 (-)	400 (-)	25 (-)
**3b**	400 (-)	200 (-)	200 (-)	200 (-)	400 (-)	100 (-)	100 (13)
**3c**	100 (-)	100 (-)	50 (-)	50 (-)	>400 (-)	200 (-)	100 (-)
**3d**	100 (-)	>400 (-)	>400 (-)	>400 (-)	200 (-)	50 (-)	200 (-)
**3e**	**6.25** [400]	**12.5** (30)	**6.25** [400]	**12.5** (28)	**3.12** [400] (40)	25 (23)	50 (23)
**4a**	>400 (-)	>400 (-)	>400 (-)	>400 (-)	>400 (-)	>400 (-)	>400 (-)
**4b**	>400 (-)	>400 (-)	>400 (-)	>400 (-)	>400 (-)	>400 (-)	>400 (-)
**5a**	400 (-)	>400 (-)	>400 (-)	>400 (-)	>400 (-)	>400 (-)	>400 (-)
**5b**	400 (-)	>400 (-)	>400 (-)	>400 (-)	>400 (-)	400 (-)	>400 (-)
**5c**	400 (-)	>400 (-)	>400 (-)	>400 (-)	>400 (-)	>400 (-)	>400 (-)
**6a**	>400 (-)	>400 (-)	>400 (-)	>400 (-)	>400 (-)	>400 (-)	>400 (-)
**7a**	25 (29)	25 (21)	25 (25)	50 (21)	25 (33)	50 (15)	50 (18)
**7b**	**6.25** (31)	**12.5** (17)	50 (22)	50 (21)	25 (27)	25 (28)	**6.25** (35)
**7c**[c]	50 [200] (33)	50 (29)	**12.5** [100]	50 [200] (20)	25 [200] (40)	50 (16)	**12.5** [25] (34)
Fl[d]	1 (43)	2 (32)	0.38 (39)	0.38 (40)	0.75 (40)	64[e] (16)	16[f] (12)

The highest activity against yeasts indicated by the low MIC values (≤12.5 mg/L) is shown in boldface.

(-) – The inhibition zone was not observed in the disc-diffusion method. The diameter of paper discs was 9 mm; NT – not tested. The MIC determination could not be performed, because the tested substance dissolved in DMSO precipitated after implementation into the RPMI medium.

[a] Only the MFC values ≤400 mg/L are presented.

[b] The MIC and MFC values of the substance were determined up to 100 mg/L. In the table, only the MFC values ≤100 mg/L are presented. The tested substance dissolved in DMSO precipitated after implementation into the RPMI medium at a concentration above 100 mg/L.

[c] The MIC and MFC values of the substance were determined up to 200 mg/L. In the table, only the MFC values ≤200 mg/L are presented. The tested substance dissolved in DMSO precipitated after implementation into the RPMI medium at a concentration above 200 mg/L.

[d] FL, fluconazole was used as a reference antifungal agent; the diameter of the commercial disc containing 0.025 mg of fluconazole was 6 mm; the MIC value of fluconazole was determined by the Etest method 95].

[e] The ellipse was visible pointing the MIC value 64 mg/L, however, with macro-colonies up to concentration ≥256 mg/L. In accordance with the recommendations for Etest method, the MIC value of fluconazole against *C. krusei* can be also interpreted as ≥256 mg/L [95,96]. *C. krusei* is intrinsically resistant to fluconazole.

[f] The ellipse was visible pointing the MIC value 16 mg/L, with colonies up to concentration ≥256 mg/L. There are no recommendations for Etest method interpretation of the MIC value of fluconazole against *S. cerevisiae*. The obtained MIC 16 mg/L is in line with the published results [97].

Table S4. The antibacterial activity of studied compounds against β-lactamase-producing Gram-negative strains.

Agent tested	Diameter of inhibition zone (mm) around discs with 0.03/0.1/0.3 mg of a tested agent			
	K. pneumoniae **ATCC BAA 1705 KPC(+)**	***P. aeruginosa*** **MUW 700 AmpC(+)**	***E. coli*** **77 CMY-2(+)**	*K. pneumoniae* **ATCC 700603 ESBL(+)**
PBA	-/-/-	-/-/-	-/-/-	-/-/-
**1a**	-/-/-	-/-/-	-/-/-	-/-/-
**1b**	-/-/13*	-/-/12*	-/-/12*	-/-/12*
**1c**	-/-/12*	-/-/13	-/-/17*	-/-/14*
**1d**	-/-/15*	-/-/12*	-/-/13*	-/-/14*
**1e**	-/-/15*	-/-/14	-/-/18*	-/-/14*
**1f**	-/-/16	-/-/12*	-/-/15*	-/-/18*
**1g**	-/-/-	-/-/-	-/-/-	-/-/-
**1h**	-/-/-	-/-/-	-/-/-	-/-/-
**1i**	-/-/15*	-/-/12*	-/-/16*	-/-/13*
**1j**	-/-/-	-/-/14*	-/-/-	-/-/11*
**1k**	-/-/-	-/-/-	-/-/-	-/-/-
**1l**	-/-/-	-/-/-	-/-/-	-/-/-
**2a**	-/-/-	-/-/-	-/-/-	-/-/-
**2b**	-/-/-	-/-/11*	-/-/13	-/-/-
**2c**	-/-/14*	-/-/11*	-/-/15*	-/-/13*
**2d**	-/-/11*	-/-/12*	-/-/15*	-/-/11*
**2e**	-/-/-	-/-/-	-/-/-	-/-/-
**2f**	-/-/-	-/-/-	-/-/-	-/-/-
**2g**	-/-/-	-/-/-	-/-/-	-/-/-
**3a**	-/-/-	-/-/-	-/-/-	-/-/-
**3b**	-/-/-	-/-/-	-/-/-	-/-/-
**3c**	-/-/-	-/-/-	-/-/-	-/-/-
**3d**	-/-/-	-/-/-	-/-/-	-/-/-
**3e**	-/-/-	-/-/-	-/-/-	-/-/-
**4a**	-/-/-	-/-/-	-/-/-	-/-/-
**4b**	-/-/-	-/-/-	-/-/-	-/-/-
**5a**	-/-/-	-/-/-	-/-/-	-/-/-
**5b**	-/-/-	-/-/-	-/-/-	-/-/-
**5c**	-/-/-	-/-/-	-/-/-	-/-/-
**6a**	-/-/-	-/-/-	-/-/-	-/-/-
**7a**	-/12*/17	-/11*/12	-/13*/18	-/18*/18
**7b**	-/-/14	-/-/-	-/12*/14	-/-/13*
**7c**	-/-/-	-/-/-	-/-/-	-/-/-

(-) – indicates no inhibition zone; PBA – phenylboronic acid.
* colonies within the inhibition zone. The diameter of the outer zone edge is presented, according to EUCAST recommendations for the fosfomycin testing against *E. coli* [98].

Table S5. The antibacterial activity of tested agents against β-lactamase-producing Gram-negative strains.

	MIC in mg/L					
**Agent tested***	***E. coli* 76 KPC-2(+)**	***E. coli* 77 CMY-2(+)**	***K. pneumoniae* ATCC BAA-1705 KPC-2(+)**	***K. pneumoniae* 81 KPC-3(+)**	***K. pneumoniae* 83 KPC-3(+), CTX-M-3(+)**	***P. aeruginosa* MUW 700 AmpC(+)**
PBA	>400	>400	>400	>400	>400	>400
**1a**	>400	>400	>400	>400	>400	>400
**1b**	400	400	>400	>400	>400	>400
**1c**	200	200	200	200	200	400
**1d**	400	400	400	400	400	>400
**1e**	100	100	200	200	200	200
**1f**	100	100	100	100	100	>400
**1i**	100	100	200	200	200	>400
**2a**	>400	>400	>400	>400	>400	>400
**2b**	400	400	>400	>400	>400	>400
**2c**	200	200	400	200	200	>400
**2d**	200	200	400	400	400	>400
**2e**	>400	>400	>400	>400	>400	>400
**2f**	>400	>400	>400	>400	>400	>400
**2g**	>400	>400	>400	>400	>400	>400
**3a**	400	400	400	400	400	400
**3b**	>400	>400	>400	>400	>400	>400
**3c**	>400	>400	>400	>400	>400	>400
**3d**	>400	>400	>400	>400	>400	>400
**3e**	>400	>400	>400	>400	>400	>400
**4a**	>400	>400	>400	>400	>400	>400
**4b**	>400	>400	>400	>400	>400	>400
**5a**	>400	>400	>400	>400	>400	>400
**5c**	>400	>400	>400	>400	>400	>400
**7a**	100	100	100	200	100	400
**7c**	>200	>200	>200	>200	>200	>200

PBA – phenylboronic acid.

* MICs of agents **1g**, **1h**, **1j**, **1k**, **1l**, **5b**, **6a**, and **7b** for β-lactamase-producing Gram-negative strains were not determined since those compounds did not show any β-lactamase inhibitory activity at high concentration, according to the results of the CDTs test presented in Table 1. Thus, they were not qualified for β-lactamase inhibitory activity testing at low concentrations.

Table S6. The MIC values of antibiotics alone and in combination with studied compounds against standard and clinical strains of Gram-negative rods producing various classes of β-lactamases.

Agent tested*	MICs (mg/L) of antibiotics alone or in combination with 16/8/4 mg per L of the tested agent						
	K. pneumoniae **ATCC BAA-1705 KPC-2(+)**	***E. coli 76*** **KPC-2(+)**	***K. pneumoniae*** **81 KPC-3(+)**	***K. pneumoniae*** **83 KPC-3(+), CTX-M-3(+)**	***E. coli*** **77 CMY-2(+)**	***P. aeruginosa*** **MUW 700 AmpC(+)**	***P. aeruginosa*** **1204 VIM(+)**
	**MEM**	**MEM**	**MEM**	**MEM**	**CAZ**	**CAZ**	**MEM**
without agent	32	64	16	32	64	128	64
PBA	**8**/16/16	**16/16/16**	8/8/8	**8/8**/16	**8/16**/32	**8/8/16**	64/64/64
**1a**	**4/8**/16	**8/16**/32	**1/2**/8	**4**/16/16	**8/16**/32	**8/16/32**	64/64/64
**1b**	**8**/16/16	**16/16**/32	**1/4**/8	**8**/16/32	**4/8/16**	**16/16/32**	64/64/64
**1c**	16/32/32	32/32/64	8/16/16	32/32/32	**4/8/16**	**16/32**/64	64/64/64
**1d**	**8**/16/16	**16**/32/32	**1/4**/8	**8**/16/32	**8/16/16**	**32**/64/64	64/64/64
**1e**	**8**/16/16	**16**/32/32	**4/8**/8	**8**/16/32	**2/4/8**	**32/32**/64	64/64/64
**1f**	16/32/32	32/64/64	8/16/16	16/32/32	**4/8/16**	**32**/64/64	64/64/64
**1i**	32/32/32	64/64/64	16/16/16	32/32/32	32/32/32	64/64/64	64/64/64
**2a**	16/16/16	32/32/64	8/8/16	**4**/16/16	**8/16/16**	**16/16/32**	64/64/64
**2b**	16/16/16	32/32/32	8/8/16	**8**/16/16	**4/8/16**	**32/32**/64	64/64/64
**2c**	32/32/32	64/64/64	16/16/16	16/16/32	**8/8/16**	**16/32/32**	64/64/64
**2d**	32/32/32	64/64/64	16/16/16	16/16/16	**4/8/16**	**32/32**/64	64/64/64
**2e**	32/32/32	64/64/64	16/16/16	32/32/32	32/32/32	128/128/128	64/64/64
**2f**	32/32/32	64/64/64	8/16/16	16/16/16	32/64/64	64/64/128	64/64/64
**2g**	32/32/32	64/64/64	16/16/16	32/32/32	32/64/64	64/64/128	64/64/64
**3a**	**4/8**/16	**4/8/16**	**0.5/1/2**	**0.5/2/8**	**16**/32/32	**32**/64/128	64/64/64
**3b**	16/32/32	32/64/64	8/8/16	16/16/16	**16**/32/32	**32**/64/128	64/64/64
**3c**	16/16/16	**16**/32/32	**2/2/4**	**4/8/8**	32/32/32	128/128/128	64/64/64
**3d**	32/32/32	64/64/64	16/16/16	32/32/32	32/32/64	128/128/128	64/64/64
**3e**	32/32/32	64/64/64	8/16/16	16/16/16	64/64/64	128/128/128	64/64/64
**4a**	16/32/32	64/64/64	8/8/8	16/16/32	64/64/64	128/128/128	64/64/64
**4b**	16/16/32	32/32/64	**4**/8/8	16/16/32	**16**/32/32	**16/32**/64	64/64/64
**5a**	32/32/32	64/64/64	16/16/16	16/16/16	**16**/32/32	**16/32**/64	64/64/64
**5c**	32/32/32	64/64/64	16/16/16	16/16/16	**16**/32/32	**32**/64/64	64/64/64
**7a**	**8**/16/16	**16**/32/32	**1/4/4**	**4**/16/16	**2/4/8**	**8/16/32**	64/64/64
**7c**	16/32/32	64/64/64	8/16/16	32/32/32	64/64/64	128/128/128	64/64/64

MEM - meropenem; CAZ- ceftazidime; PBA - phenylboronic acid; The significant decreases (at least a 4-fold) in the antibiotic MIC values after the addition of a tested agent are shown in boldface.

* Agents **1g**, **1h**, **1j**, **1k**, **1l**, **5b**, **6a**, and **7b** were not tested in combinations with antibiotics against β-lactamase-producing Gram-negative strains due to negative results in the combination disc tests.

Table S7. The effect of agents **1a**, **2a** and **3a** on the activity of KPC-3 in the purified protein extract from *E. coli* 82 TR(pl 81) cells, visualized by the nitrocefin hydrolysis test.

Agent concentration in mg/L	Relative absorbance[1] (% Relative absorbance)[2] [Reduction of relative absorbance in %][3]			
	**PBA[4]**	**1a**	**2a**	**3a**
**1st repetition**				
0	1.986 (100%)	1.832 (100%)	1.866 (100%)	1.912 (100%)
4	1.093 (55%) [45%]	1.348 (74%) [26%]	1.605 (86%) [14%]	1.451 (76%) [24%]
8	1.117 (56%) [44%]	1.201 (66%) [34%]	1.528 (82%) [18%]	1.249 (65%) [35%]
16	0.957 (48%) [52%]	1.076 (59%) [41%]	1.280 (69%) [31%]	1.077 (56%) [44%]
**2nd repetition**				
0	2.007 (100%)	1.762 (100%)	1.823 (100%)	1.973 (100%)
4	1.087 (54%) [46%]	1.320 (75%) [25%]	1.649 (90%) [10%]	1.436 (73%) [27%]
8	1.183 (59%) [41%]	1.214 (69%) [31%]	1.520 (83%) [17%]	1.305 (66%) [34%]
16	0.975 (49%) [51%]	0.998 (57%) [43%]	1.377 (76%) [24%]	1.090 (55%) [45%]
**3rd repetition**				
0	2.07 (100%)	1.826 (100%)	1.851 (100%)	1.968 (100%)
4	1.232 (60%) [40%]	1.308 (72%) [28%]	1.595 (86%) [14%]	1.174 (60%) [40%]
8	1.165 (56%) [44%]	1.079 (59%) [41%]	1.499 (81%) [19%]	1.279 (65%) [35%]
16	0.933 (45%) [55%]	1.066 (58%) [42%]	1.274 (69%) [31%]	1.114 (57%) [43%]

[1] The presence of β-lactamases in the purified protein extract with and without tested agents was assessed by measurement of the rates of nitrocefin hydrolysis as relative absorbance at 486 nm. The level of measured absorbance indicating β-lactamase activity.

[2] Percentage of the relative absorbance measured for the purified protein extract with tested agent in comparison to the absorbance determination without this agent.

[3] Reduction of the relative absorbance level was calculated as the difference in the relative absorbance between the positive control and the sample each concentration of tested inhibitor and expressed as a percentage.

[4] PBA (phenylboronic acid) was used as the reference β-lactamase inhibitor.

3. Cytotoxic activity

Table S8. Viability of MRC-5 cells (% of viable cells ± SD) after 72 h-treatment with the studied compounds. The results were calculated from the MTT-based assay data.

Compound	Concentration of compound [mg/L]		
	**12.5**	**25**	**50**
**1a***	112.8±12.5	103.2±11.8	89.9±11.4
**1b**	140.4±6.7	140.1±8.3	117.0±1.3
**1c**	111.5±3.8	102.8±3.0	96.7±3.3
**1d**	111.6±1.1	104.1±8.9	83.3±1.2
**1e**	107.1± 3.3	105.0±10.0	116.6±4.6
**1f**	97.4±2.2	89.9±1.9	78.1±0.9
**1g**	104.2±8.2	107.4±9.2	98.7±5.8
**1h**	101.3±4.3	104.1±5.0	98.8±3.2
**1i**	122.3±0.0	113.9±4.8	101.4±0.9
**1j**	125.3±2.8	117.4±1.2	88.6±3.5
**1k**	84.3±0.9	74.2±3.5	28.9±1.0
**1l**	109.5±1.6	114.4±1.8	105.1±7.8
**2a**	94.4±2.2	75.2±10.3	73.1±13.6
**2b**	108.0±1.1	87.9±1.7	73.8±1.9
**2c**	89.1±0.5	83.6±2.7	63.8±2.5
**2d**	107.1±7.6	88.0±4.3	75.2±9.4
**2e**	96.6±6.4	84.4±4.8	63.1±1.4
**2f**	103.3±8.8	92.1±0.9	76.2±0.7
**2g**	111.3±9.7	117.3±3.7	103.4±5.1
**3a***	109.6±9.3	106.3±10.6	76.2±4.2
**3b**	98.1±6.1	91.2 ± 13.5	87.1 ± 8.0
**3c**	97.1±6.9	105.5±4.5	82.3±12.9
**3d**	91.3±7.4	100.0 ±7.7	77.7±15.5
**3e**	98.8±25.6	92.9±2.7	64.4±0.4
**4a**	101.9±6.9	84.4±6.4	87.9±8.5
**4b**	114.0±1.8	106.4±0.1	97.3±13.6
**5a**	88.6±9.2	87.1±1.6	79.2±5.1
**5b**	101.1±7.7	110.3±2.7	87.3±13.6
**5c**	102.5±3.4	97.9±1.4	85.6±0.7
**6a**	138.3±3.6	133.5±0.7	127.2±2.6
**7a**	83±2.9	79.9±3.7	67.3±3.1
**7b**	48.9±3.8	6.4±1.4	0.9±0.7
**7c**	56.4±3.0	6.6±5.1	0±0.1
PBA	81.5 ± 3.7	79.1 ± 4.4	58.7 ± 4.3

* the data were obtained for the following concentrations of compound: 16, 32 and 64 mg/L. PBA (phenylboronic acid) was used as the reference β-lactamase inhibitor.

4. Molecular modeling and hybrid QM/MM simulations

4.1. Molecular docking

In molecular docking, we paid attention to a few evaluation parameters. The most important one was the distance between the O atom of the Ser70 OH group and the B atom of the ligand ($d_{Ser70(O)–B}$) because it describes a ligand ability to form a protein-ligand dative covalent bond. Next, we assessed the occupation of each cavity by each docking mode. Published results [69] point that the most critical are S1 and S2 cavities where the catalytic amino acids are located (Ser70, Lys73, Glu166, see Figure 3). Finally, we also included in the analysis the estimated binding free energy. However, we omitted the other parameters generated by Molecular Operating Environment [84]. In general, most of the modes in an anionic form did not fulfil the distance criterion. For an anionic form of each ligand, most modes were directed to the O atom of the Ser70 hydroxyl group by the sp^3 hybridized boronate group. Since three hydroxyl groups are attached to the B atom, a protein-ligand dative bond cannot form. Thus, the covalent bond can occur solely for the sp^2 hybridized boron atom. However, even though the docking modes positioned the boron atom in the sp^2 hybridization in the proper way, still it was too far to pass the geometric criteria. Therefore we did not describe such forms further.

From all generated conformations of PBA, we chose one that scored –3.78 kcal/mol. The boron atom was 3.46 Å away from the O atom of the Ser70 OH group and occupied the S1 and S1′ cavities. For **1a**, we chose three respective binding modes (B–O distance from 3.55–4.28 Å). However, they occupy only the S1 or S2 cavity. The *para* substitution with two $B(OH)_2$ groups prevents simultaneous occupation of the S1 and S2 cavities. Thus, we could describe only two possible binding modes (Fig. S15): the first one is an arrangement where the ligand fits along the active site, and its ring is directed perpendicular to the Trp105 rings (Fig. S15A). This arrangement is not ideal because at the entrance to the active site there is a conflict with Trp105, so the ligand cannot rotate to the orthogonal arrangement of the B group to Ser70. The second mode involves the parallel conformation of the ligand to Trp105 allows reaching Ser70, however, half of the ligand protrudes from the active site (Fig. S15B). For **2a**, all three chosen binding

modes occupied the S1 and S2 cavities simultaneously and the B atoms were quite close to the O atom of the Ser70 OH group ($d_{Ser70(O)–B}$ = 3.33–3.45 Å). Compound **3a** is the *ortho* isomer which, besides the classical anionic form **3a_II**, can also potentially convert to the neutral cyclic semi-anhydride form **3a_III** and the corresponding anionic forms (**3a_IV**, **3a_V**). For the parent form **3a_I** we selected three binding modes, which reached the shortest value of $d_{Ser70(O)–B}$ from all analyzed ligands (from 3.10 Å to 3.28 Å). We also chose the three most interesting binding modes of **3a_III** with distance range similar to that for **3a_I**. All chosen modes occupy the S1 and S2 cavities. Interestingly, from all generated **3a_IV** binding modes, we distinguished one in which the sp^2-hybridized B atom occupied the S1 cavity ($d_{Ser70(O)–B}$ = 3.30 Å) and the molecule covered almost the entire active site excluding the S2′ cavity. The structure **3a_V** was not taken into account in simulations due to the lack of any boronic group in the sp^2 hybridization. Finally, 14 binding modes were qualified for further MD simulations.

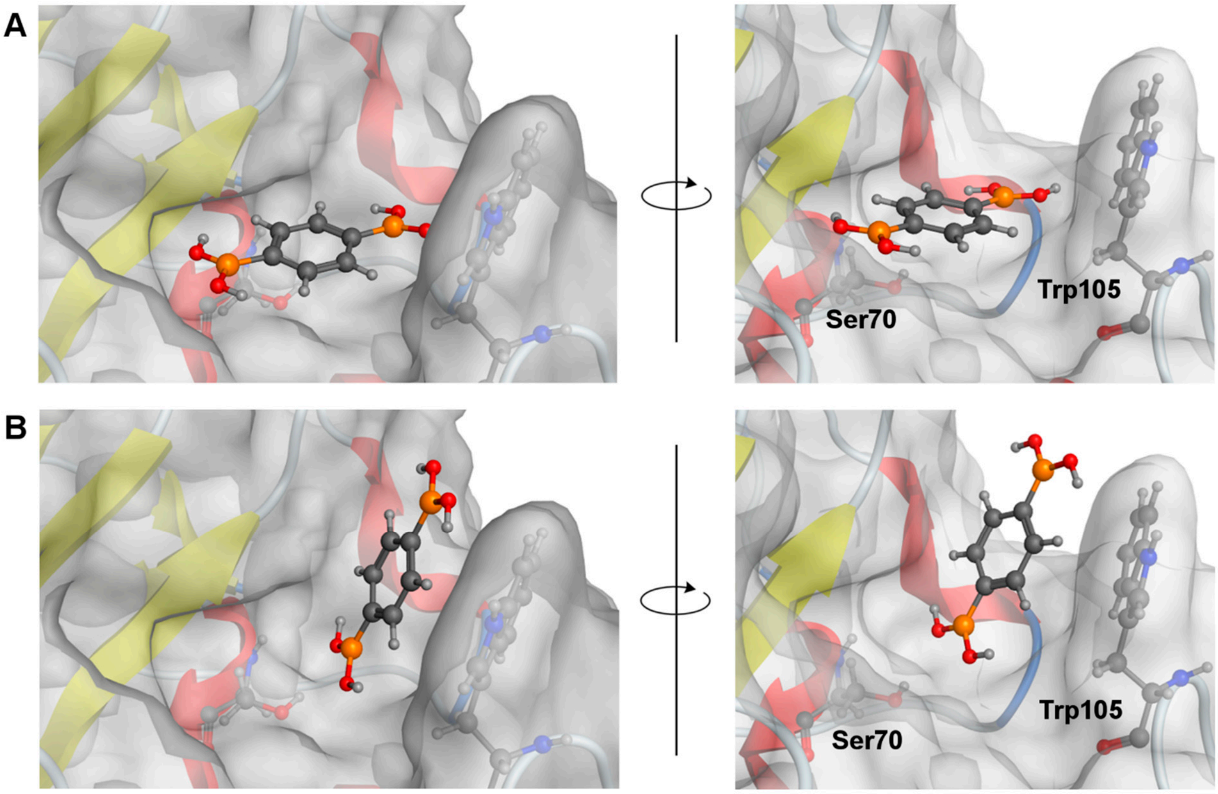

Figure S15. Two types of the binding modes of **1a**: A) The perpendicular configuration of ligand to Trp105, B) The parallel configuration of ligand to Trp105.

4.2. Molecular dynamics

Based on results of performed 14 MD simulations, we concluded that each system reached the thermodynamic equilibrium after 1.1 ns. However, single modes of **2a** and **3a_I** pulled out from the active site to the solvent. The other 12 modes occupied the KPC-2, which indicates that they were well-fitted to the active site during simulations. The results of the most stable fragment of the entire trajectory (time range from 1.8 to 2.0 ns) were analyzed Notably, during MD simulation, after each 0.5 ps, an arrangement was saved. Thus, for each ligand in the considered trajectory range, we obtained 500 arrangements to analyze. Such a pool of conformations allowed to estimate the ability to form the covalent bond between the O atom of the Ser70 OH group and the B atoms of studied diboronic acids. We treated only the arrangements with $d_{\mathrm{Ser70(O)–B}}$ = 3.0 Å. Just such a distance can allow for initiating the competitive inhibition [69]. For the chosen PBA mode, the 40.4% arrangements of the analyzed fragment of trajectory have the ability to form a covalent protein-ligand bond. It means that the ligand was positioned in a configuration where the B atom in sp^2 hybridization is closely located and directed to the O atom of the Ser70 OH group. For the rest of the simulated complexes such a propensity was also observed. Two docking modes of **1a** show the potential to accomplish the competitive inhibition but at different arrangements. We observed the same situation for **2a** and **3a_III**. For these systems, we chose docking modes which had a lower average distance estimation between the O atom of the serine OH group and the B atom. Thus, for **1a** only 2.6% of all arrangements can form a covalent bond. For **2a**, only 1.2% arrangements possess such ability in the entire analyzed fragment of trajectory. All forms of **3a** can create the possible protein-ligand covalent bond. The most promising form of **3a** is **3a_III**, where 22.4% of arrangements have $d_{\mathrm{Ser70(O)–B}} \leq 3.0$ Å. For **3a_I** and **3a_IV**, the percentage of effective arrangements is less, *i.e.*, 11.0 and 6.8%, respectively. In addition, from the analyzed part of the trajectory, an arrangement with the minimal value $d_{\mathrm{Ser70(O)–B}}$ was extracted. Such arrangement can be treated as the most promising binding mode and starting point for quantum mechanics/molecular mechanics simulations (QM/MM).

4.3. Quantum-mechanics/molecular mechanics simulations

QM/MM simulations were performed with eight repetitions (4 repetitions for each starting structure). For several simulations, we successfully detected a formation of protein-ligand covalent bond whose mechanism is in agreement with previous results [69]. For docking modes of PBA simulations did not result in covalent binding (0/4). The same results we observed for **1a**, **2a**, and **3a_I**. Interestingly, for modes of **3a_III** and **3a_IV**, we distinguished the formation of protein-ligand covalent bond – three times for **3a_III** and four times for **3a_IV**. In an arrangement from the molecular dynamics for PBA, we observed twice the occurrence of such a reaction. The ability to form protein-ligand covalent bonds was also identified for the remaining analyzed ligands. We observed twice an interesting inhibitory mechanism for **1a**, **2a**, and **3a_I**. The single event was identified for **3a_III**. The case of **3a_IV** is very interesting as the covalent bond was formed between the O atom of the Ser70 OH group and the B atom in all repetitions. Such a significant difference of the results generated by the QM/MM of docking modes and by the QM/MM of extracted arrangement from MD is not surprising. Comparing $d_{Ser70(O)–B}$, we see that the minimal value was decreased during the MD simulation. For PBA, it was 2.70 Å, for **1a** – 2.80 Å, for **2a** – 2.87 Å. The best results were observed for **3a**, where the minimal $d_{Ser70(O)–B}$ was the shortest (**3a_I** – 2.73 Å**, 3a_III** – 2.66 Å, **3a_IV** – 2.82 Å). Noteworthy, the value of $d_{Ser70(O)–B}$ should be in the order of 3 Å, or slightly less before the chemical bond is formed [69]. It is well observed in our results, where all analyzed ligands shifted (the most for **1a** – 0.94 Å and PBA – 0.76 Å, **2a** – 0.58 Å, **3a_I** – 0.55 Å, **3a_III** – 0.56 Å, the least for **3a_IV** – 0.48 Å). Such observed rearrangements in the closest area of the O atom of the Ser70 OH group increase the probability of forming the protein-ligand covalent bond.

Another important factor for the formation of the protein-ligand covalent bond is the ligand association angle [69]. These studies suggest that the adequate orientation of the BLI should be specified by the angle of ca. 90° between C–B and HO–C bonds [69]. It allows for the interaction of the empty 2p orbital of the B atom with the lone pair of the O atom of the Setr70 OH group [69]. During the formation of the dative covalent bond, the B atom changes its hybridization from sp^2 to sp^3, resulting in increase of the

ligand association angle to around 110° [69]. We observed such angles in the all repetitions, where the competitive inhibition took place. All presented results suggest that all of the analyzed diboronic inhibitors can form a covalent bond with KPC-2, and we can identify them as BLI. In addition, the obtained trajectories allowed us to distinguish the moments when the most important steps of mechanism of protein-ligand covalent bond (Table S9).

Table S9. The simulation times to reach the most important steps in mechanism of protein-ligand covalent bond formation in the most promising BLIs, calculated from the beginning of the process. Description of the transfer processes follows the IUPAC nomenclature indicating which atoms are engaged. All times are given in ps.

Ligand	Lys73:N^6-H →	Ser70:O^3-H → Lys73:N^6	LIG:B^1 → Ser70:O^3
PBA	0.42	10.47	10.59
**1a**	0.70	8.11	8.49
**2a**	0.42	16.85	17.56
**3a_I**	0.52	27.14	27.31
**3a_III**	0.47	9.96	10.63
**3a_IV**	0.41	0.95	1.46

Such steps are consistent with the already published mechanism, observed for BLI inhibitors [69]. The time needed for proton transfer from Lys73 to Glu166 agrees with the determined and published mechanism of inhibition [69]. However, the other steps are related with the ligand structure or with the isomerism (in our case, the positions of two boronic groups). Thus, the ability of BLI to form the dative covalent bond should be estimated as the probability of its formation under favorable conditions ($P_{FAVORABLE}$). For **3a_I** and **3a_III** we observed interesting transformation of these compounds into the **3a_V** form, with the protein-ligand covalent bond instead one of the OH group. For **3a_I**, after a successful covalent binding, the internal cyclization of a compound was observed in a half of the repetitions. As a result, the second B atom changes its hybridization to sp^3 (Fig. S16). It is an immediate reaction, *i.e.*, it proceeds within 1.5 ps from the moment of formation of a covalent bond with Ser70. Such a transformation

occurs also for **3a_III**. Such a change was identified in 2 out of 8 repetitions. Due to the necessity of the presence of two H_2O molecules, further transformations of **3a_III** proceed much later than the moment of forming the covalent bond with Ser70. After 29.48 ps, proton transfer from Glu166 to the O atom built in the 5-element ring of the BLI was observed. After 0.05 ps the subsequent reactions occurred. They involved a proton transfer from the first H_2O molecule to Glu166, a proton transfer from the first H_2O molecule to the second one, and a nucleophilic addition of the second H_2O molecule to the sp^{2-} hybridized B atom (Fig. S17).

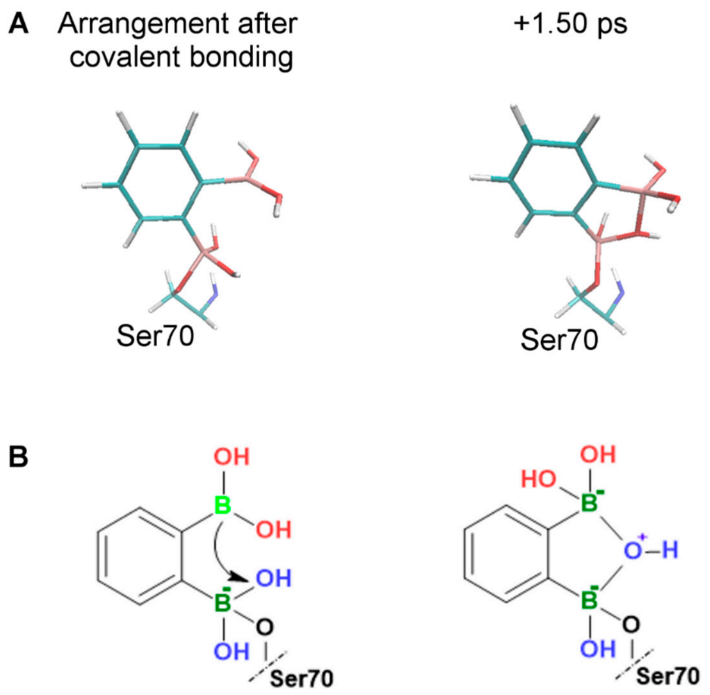

Figure S16. Transformation of **3a_I** to **3a_V**. A) The arrangement before and after the intramolecular cyclization. B) Transformation mechanism. Bright/dark green color indicates sp^2/sp^3 hybridization of the B atom. For clarity, the O and H atoms are marked with different colors.

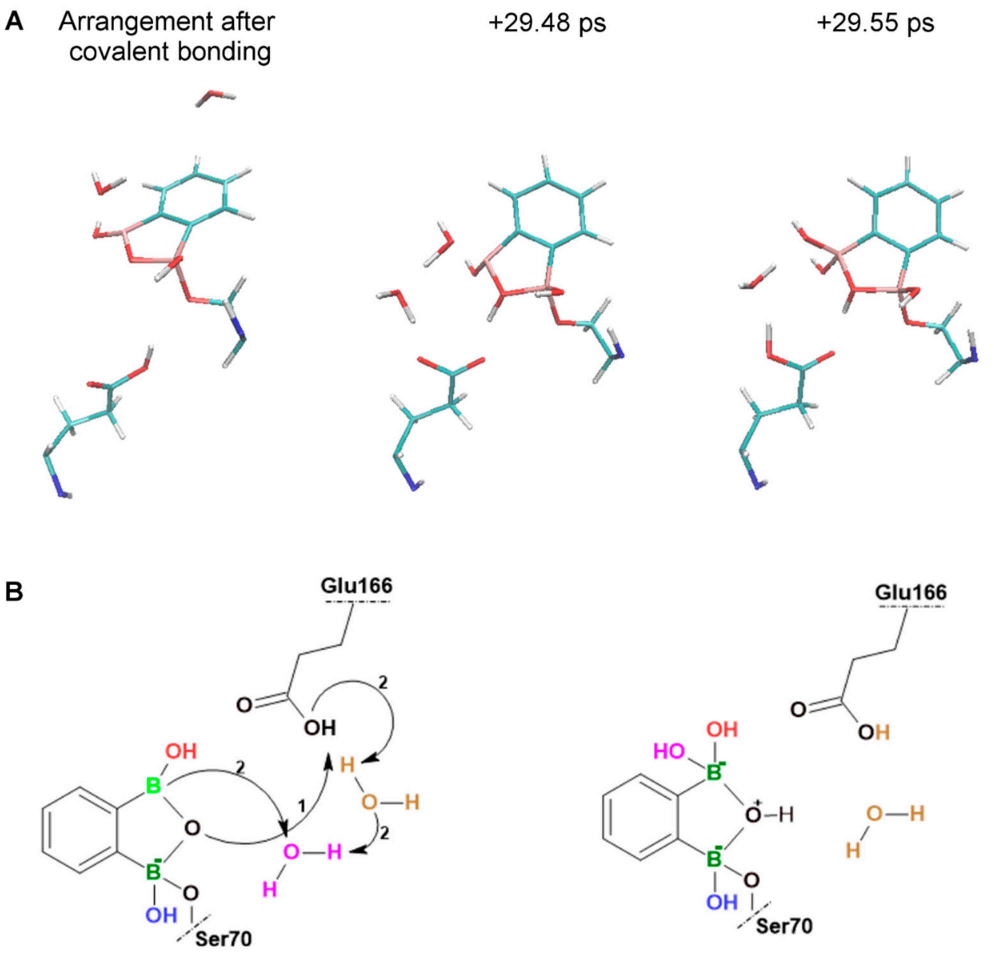

Figure S17. Transformation of **3a_III** to **3a_V**. A) The arrangement before, during, and after the reaction. B) Transformation mechanism. The numbers at the arrows indicate the sequence of events. Bright/dark green color indicates sp^2/sp^3 hybridization of the B atom. For clarity, the O and H atoms are marked with different colors.

5. References

69. Charzewski, Ł.; Krzyśko, K.A.; Lesyng, B. Exploring covalent docking mechanisms of boron-based inhibitors to class A, C and D β-lactamases using time-dependent hybrid QM/MM simulations. Front. Mol. Biosci. 2021, 8, 633181, DOI:https://doi.org/10.3389/fmolb.2021.63318175.

75. Clinical and Laboratory Standards Institute (CLSI). Methods for dilution antimicrobial susceptibility tests for bacteria that grow aerobically. Approved Standard, CLSI Dokument M07-A9. 9th ed. CLSI: Wayne, PA, USA 2012.

84. Chemical Computing Group ULC, Molecular Operating Environment (MOE). 1010 Sherbrooke St.West 910, Montreal, QC, Canada, 2018. Available online: https://www.chemcomp.com/Products.htm (accessed on 1 October 2023).

94. Ren, Y.; Bazan, G.C. Trifluoromethyl-substituted conjugated oligoelectrolytes. Chemistry 2010, 16, 11028-11036, doi:https://doi.org/10.1002/chem.201000885.

95. ETEST. Application guide. Available online: https://www.biomerieux-usa.com/sites/subsidiary_us/files/supplementary_inserts_-_16273_-_b_-_en_-_eag_-_etest_application_guide-3.pdf (accessed on 1 October 2023)

96. Espinel-Ingroff, A. Etest for antifungal susceptibility testing of yeasts. Diagn. Microbiol. Infect. Dis. 1994, 19, 217-220, doi:10.1016/0732-8893(94)90034-5.

97. Pfaller, M.A.; Bale, M.; Buschelman, B.; Lancaster, M.; Espinel-Ingroff, A.; Rex, J.H.; Rinaldi, M.G. Selection of candidate quality control isolates and tentative quality control ranges for in vitro susceptibility testing of yeast isolates by National Committee for Clinical Laboratory Standards proposed standard methods. J. Clin. Microbiol. 1994, 32, 1650-1653, doi:10.1128/jcm.32.7.1650-1653.1994.

98. European Committee on Antimicrobial Susceptibility Testing. Reading guide. EUCAST disk diffusion method for antimicrobial susceptibility testing. Document version 9.0. Available online: https://www.eucast.org/fileadmin/src/media/PDFs/EUCAST_files/Disk_test_documents/2022_manuals/Reading_guide_v_9.0_EUCAST_Disk_Test_2022.pdf (accessed on 1 October 2023)
